# Diversification and historical demography of *Rhampholeon spectrum* in West-Central Africa

**DOI:** 10.1371/journal.pone.0277107

**Published:** 2022-12-16

**Authors:** Walter Paulin Tapondjou Nkonmeneck, Kaitlin E. Allen, Paul M. Hime, Kristen N. Knipp, Marina M. Kameni, Arnaud M. Tchassem, LeGrand N. Gonwouo, Rafe M. Brown

**Affiliations:** 1 Department of Ecology and Evolutionary Biology, University of Kansas, Lawrence, Kansas, United States of America; 2 Biodiversity Institute, University of Kansas, Lawrence, Kansas, United States of America; 3 Laboratory of Zoology, Faculty of Science, University of Yaoundé I, Yaoundé, Cameroon; Shiv Nadar University, INDIA

## Abstract

Pygmy Chameleons of the genus *Rhampholeon* represent a moderately diverse, geographically circumscribed radiation, with most species (18 out of 19 extant taxa) limited to East Africa. The one exception is *Rhampholeon spectrum*, a species restricted to West-Central African rainforests. We set out to characterize the geographic basis of genetic variation in this disjunctly distributed *Rhampholeon* species using a combination of multilocus Sanger data and genomic sequences to explore population structure and range-wide phylogeographic patterns. We also employed demographic analyses and niche modeling to distinguish between alternate explanations to contextualize the impact of past geological and climatic events on the present-day distribution of intraspecific genetic variation. Phylogenetic analyses suggest that *R*. *spectrum* is a complex of five geographically delimited populations grouped into two major clades (montane vs. lowland). We found pronounced population structure suggesting that divergence and, potentially, speciation began between the late Miocene and the Pleistocene. Sea level changes during the Pleistocene climatic oscillations resulted in allopatric divergence associated with dispersal over an ocean channel barrier and colonization of Bioko Island. Demographic inferences and range stability mapping each support diversification models with secondary contact due to population contraction in lowland and montane refugia during the interglacial period. Allopatric divergence, congruent with isolation caused by geologic uplift of the East African rift system, the “descent into the Icehouse,” and aridification of sub-Saharan Africa during the Eocene-Oligocene are identified as the key events explaining the population divergence between *R*. *spectrum* and its closely related sister clade from the Eastern Arc Mountains. Our results unveil cryptic genetic diversity in *R*. *spectrum*, suggesting the possibility of a species complex distributed across the Lower Guinean Forest and the Island of Bioko. We highlight the major element of species diversification that modelled today’s diversity and distributions in most West-Central African vertebrates.

## Introduction

**Fig 1:**
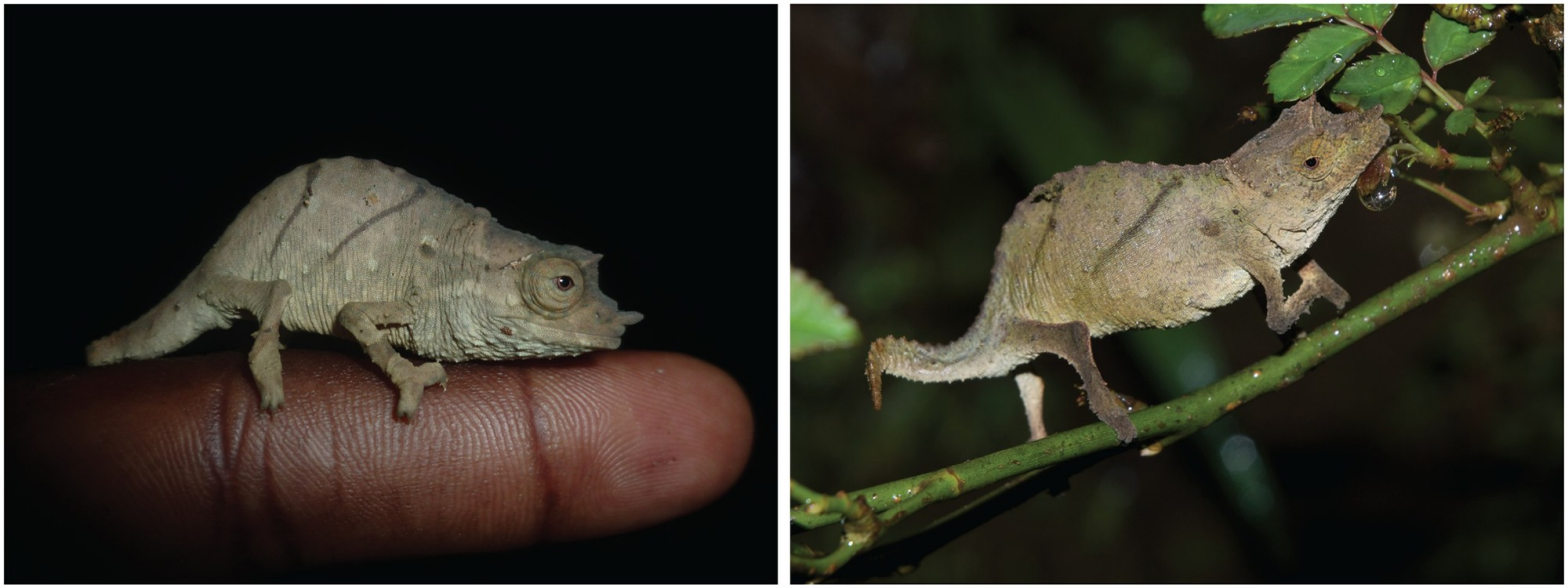
*Rhampholeon spectrum*. (Left) Male from Ekona Lelu, Mt. Cameroon. (Right) Female from Mt. Kupe. Photograph credit Luke Welton (right image).

**Fig 2:**
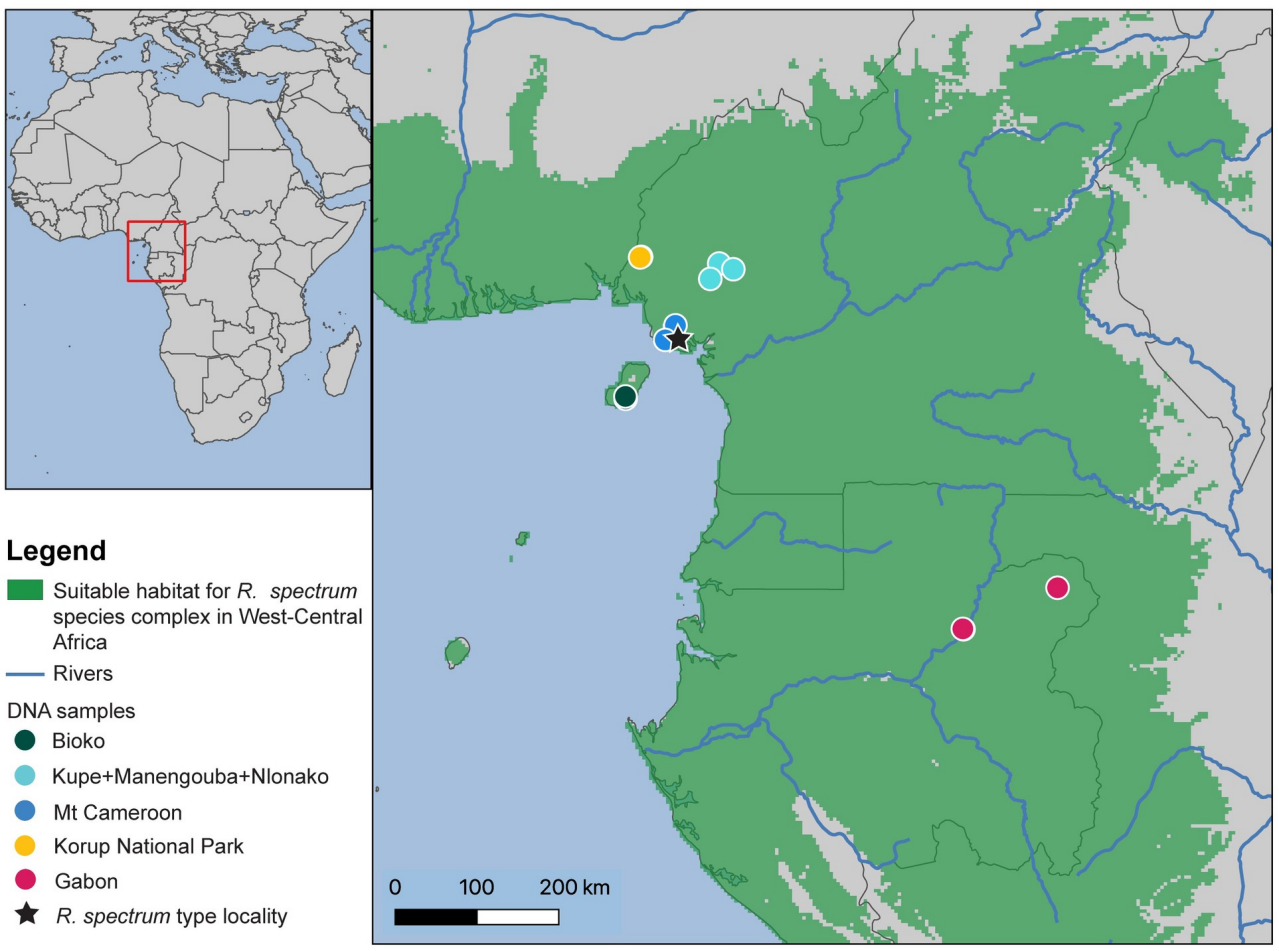
Map of suitable habitat for *Rhampholeon spectrum* species complex in West-Central Africa. Present-day suitable habitat was generated using kuenm. Sampling localities are indicated by various-shaded circles.

Pygmy, or Leaf Chameleons, genus *Rhampholeon*, occurs in lowland, sub-montane, and montane forest patches from West to East Africa [1]. This genus contains 19 described species [2], with 18 taxa endemic to East Africa [3]. Matthee et al. [4] partitioned the members of the genus *Rhampholeon* into three subgenera: *Rhampholeon*, *Rhinodigitum*, and *Bicuspis*. *Rhampholeon* species are forest leaf-litter specialists with notably reduced vagility [1] and despite the presence of suitable migration corridors, they are unlikely to disperse over long distances [4]. As a possible consequence of this ecological characterization, most Pygmy Chameleon species presently are considered endemic to the single mountains or isolated forest patches from where they were originally described [5–7]. *Rhampholeon* (*Rhampholeon*) *spectrum* [8] (Fig 1), originally described from Bonjongo South of Mount Cameroon, is the type species for the genus *Rhampholeon* [9]. It is the only species known to occur in West-Central Africa (also referred by some authors as the Lower Guinean Forest) and exhibits an atypical disjunct distribution from the east African sister clade (Fig 2) which renders it of particular interest to biogeographers [4].

Three major geographic features surround the current distribution range of *R*. *spectrum*; the Niger River in the east, the Adamawa plateau in the north, and finally the Congo River in the south and the east. This distribution encompasses one of the most diverse regions of continental Africa [10]. Within the West-Central African region, Ecotones [11], river barriers [12], mountain range formation [13], and repeated expansion and contraction of forests into and out of refugia during past climatic fluctuations [14, 15] have been listed by previous studies as alternate hypothesis working together or separately as the principal drivers of species diversification in Central African rainforests [16, 17]. Therefore, geographically structured genetic variation in unrelated but co-distributed forest‐restricted species often coincides with the locations of these geographical and landscape features [18, 19] (Fig 2). However, taxonomic divisions and range boundaries among species distributed across West and Central Africa are complex and taxon-specific [20, 21], which intensifies the challenges of characterizing and distinguishing among mechanisms of evolutionary diversification via traditional phylogeographic studies. *Rhampholeon spectrum* has never been the subject of a range-wide genomic characterization of geographic variation. Due to their distribution spanning many recognized landscape features of West-Central Africa, *R*. *spectrum* is an ideal focal species to independently assess the significance of the variable Central African geographical template as part of the diversification history of this virtually unstudied endemic vertebrate lineage.

The overall objective of this work is to provide an evolutionary framework for the diversification of Pygmy Chameleons across the range of *R*. *spectrum* in West-Central Africa. With novel sampling and genome-wide data, we test competing hypotheses regarding the role of abiotic environmental factors hypothesized to have driven the evolution of forest vertebrates in the Lower Guinean Forest. The specific goals of this study are (1) to infer the first robust, comprehensive molecular phylogeny for populations referred to *R*. *spectrum*; (2) to test for congruence of the time-calibrated phylogenetic analysis with current knowledge of geological events in West-Central Africa; (3) to test for recent population admixture and its potential impact on the resulting population structure; and (4) to test of whether the geographic template and landscape features (Ecotones, river barriers, mountain range formation) have led to detectable geographically-based genetic structure in *R*. *spectrum*.

## Materials and methods

### Taxon sampling

*Rhampholeon spectrum* samples were obtained from field expeditions to Cameroon between January 2017 and August 2018, and via tissue grants from the California Academy of Science and the Museum of Comparative Zoology, Harvard University (Fig 2). Genetic sequences corresponding to three additional samples were drawn from records in GenBank and were used only in analysis of Sanger-sequenced data (S1 Table). New samples came from the following sites (number of individuals in brackets): Cameroon (Mt. Cameroon [1], Mt. Manengouba [4], Mt. Nlonako [5], Mt. Kupe [2], and Korup National Park [9]), Gabon (Ivindo [3] and Mekambo [1]), and Equatorial Guinea (Bioko Island [5]). Voucher specimens are deposited in the herpetology collection of the University of Kansas and the California Academy of Science.

This study was carried out in accordance with the Institutional Animal Care and Use Committee. The protocol was approved under the authorization number Brown AUS 158–04 of the University of Kansas. Animal euthanasia was done by injection using Tricaine Methanesulfonate (MS222). Research permit number 005/MINRESI/B00/C00/C10/C14 was granted respectively by the Ministry of Scientific Research and Innovation of Cameroon. Research permit numbers 1261/PRS/MINFOF/SG/DFAP/SDVEF/SC and CITES permits number 0723-5/P/MINFOF/SG/DFAP/SVDEF/SC/BJ were granted by the Ministry of Forestry and Wildlife of Cameroon.

### DNA extraction and sequencing

All tissue samples (liver) had been field-preserved in 95% ethanol. DNA extractions were performed using a Promega Maxwell RSC extraction robot and a modified version of the bead DNA extraction protocol from Phyletica Lab at Auburn University [22]. Two mitochondrial genes (*16S* and *ND4*) and one nuclear gene (*RAG1*) were amplified following standard protocols [23–25]. The primer pairs used for amplification of each gene are listed in S2 Table.

PCR products were sequenced at GENEWIZ. The complementary reads were *de novo* assembled and edited in Geneious Prime v2021.0.3 using default parameters. We used MAFFT v1.4.0 Multiple Alignment [26] implemented in Geneious to align the paired sequences and then concatenate sequences for each aligned marker (deposited in GenBank OP716816-OP716845 and OP734758-OP734807). Gene sequences (*16S*, *ND4*, and *RAG1*) from 17 of the 18 remaining species from the genus *Rhampholeon* and three species of *Rieppeleon* obtained from GenBank were used as outgroups. To characterize genetic divergences among species and *R*. *spectrum*’s populations, we computed the pairwise genetic distances (16S and ND4) using net sequence divergences (uncorrected *p*-distances) in MEGA 11 [27].

### Genomic data

We sequenced genome-wide anonymous nuclear markers for 28 individuals, following a modified version of the ddRADseq protocol of Peterson et al. [28]. The detailed protocol for library prep and pooling is available in the supplementary information. Library pools were combined in equimolar amounts for sequencing on one Illumina HiSeqX Lane at Novogene. We used STACKS v2.5 [29] to process the Illumina reads from the ddRAD, and then used a read-stitching approach [30] to join the first read from an Illumina read pair with the reverse complement of the second, recapitulating the original orientation of fragments in the genome.

We tested a range of assembly parameters in STACKS to optimize recovery of putative single-copy, orthologous loci, because the optimal *de novo* assembly of ddRADseq data can vary widely across taxa [31, 32]. The parameters that were modified were: (M = 2–8) the maximum number of gaps allowed between nucleotides within samples, (n = 5–15) the number of mismatches allowed in the alignment between samples when constructing the catalog of all consensus loci, (r = 50–95%) the minimum percentage of individuals in a population required to process, and (p = 1–5) the number of populations each SNP needed to be present in to be called. Parameters not mentioned above were kept at default values. The final dataset used the combination of these parameters that produced the most single nucleotide polymorphisms (SNPs) without loss in depth of coverage across loci. The assembled loci, obtained through this STACKS workflow, were aligned and concatenated in Geneious Prime.

### Phylogenetic and molecular clock analyses

#### Bayesian inference phylogenetics

We conducted Bayesian divergence-dating analyses with our concatenated mtDNA and nuclear dataset (*16S*+*ND4*+*RAG1*) partitioned by marker using BEAST 2.6.3 [33], and run on the CIPRES Science Gateway v3.3 [34]. We used bModelTest 1.2.1 [35] to average over all possible substitution models instead of selecting a single model. We implemented an uncorrelated, log-normally distributed relaxed-clock model, with an empirically estimated clock rate to allow for rate heterogeneity among lineages. To maximize calibration points, we included up to two species per chameleon genus from those available on GenBank to provide a robust representation of the family Chamaeleonidae. Fossil and secondary calibration points were used at nodes A to E (S1 Fig and S3 Table) to achieve temporal congruence with the most comprehensive time-calibrated chameleon phylogeny published to date which was based on 13 genetic markers and included nearly all chameleon species described at that time [36]. For each calibration point, we used BEAUti to build a translated log-normal distribution with an offset equal to the age of the fossil or node split. Analyses were run twice, each time for 100 million generations, and sampled every 10,000 generations. We confirmed convergence for each run separately, using TRACER 1.7 [37], after which runs were combined in LogCombiner v2.6.3, producing 20,000 trees, from which an initial 20% burn-in was discarded. TreeAnnotator v2.6.3 was used to choose the maximum clade credibility tree with the “median node heights” option from the 18,000 post-burn-in output trees [33].

#### Maximum likelihood phylogenetic inference

We used IQ-TREE v1.6.12 [38, 39] to infer maximum likelihood (ML) trees from Sanger and ddRAD multilocus data sets. For the Sanger-sequenced multilocus data set, we treated each locus as a separate partition using partition models [40] and ModelFinder [41] integrated in IQ-TREE to identify and assign the best-fit substitution model for each partition during tree inference. Our model included seven partitions: six independent partitions for each codon position of the protein-coding genes ND4, and RAG1, and a single partition for the mitochondrial gene 16S. We performed 10,000 ultrafast (UFboot) and 10,000 normal (Shimodaira-Hasegawa) bootstrap replicates to assess heuristic support for inferred clades. We considered ultrafast bootstrap support values UFboot ≥ 95 and SH-aLRT ≥ 80% to indicate strong support for monophyletic groups [42, 43].

#### Quartet inference from ddRAD-derived SNPs

We further investigated phylogenetic relationships using analyses that account for differences in the genealogical histories of individual loci. Specifically, we used the program SVDQuartets, a quartet sampling method that accounts for sequence variability owing to both mutational and coalescent variance [44]. Because SVDQuartets uses site pattern frequencies and bypasses gene tree inference and uses singular value decomposition scores [45], it has an advantage over summary-statistic-based methods for estimating species trees. Three independent runs of SVDQuartets were conducted in the program PAUP* 4.0 [44, 46] to assess topological convergence, each of which included 500 bootstrap replicates and exhaustive quartet sampling.

### Population genetic clustering and ancestry inference

A principal components analysis (PCA) was conducted in R [47] using R package Adegenet v2.1.3 [48] to visualize the population genetic clustering among individuals. These clusters were further investigated using discriminant analysis of principal components (DAPC) [48]. As a multivariate statistical method, DAPC does not make any assumptions about Hardy–Weinberg or linkage equilibrium. The function find.clusters was used to evaluate the number of population (K) values between 1 and 10 using the Bayesian information criterion (BIC), and to select the K with the lowest BIC score.

We used the likelihood-based method Structure 2.3.4 [49, 50] to identify ancestral population clusters and to investigate potential admixture between populations set using Markov Chain Monte Carlo (MCMC) simulations. Hierarchical analyses were performed for 10 runs per population K, up to a maximum of eight populations, using the admixture model with a burn-in of 100,000 iterations, followed by 10 million steps. We summarized our results using the R package POPHELPER [51] and evaluated the likely number of populations based on inspection of likelihood plots and following the Evanno method [52] implemented in POPHELPER.

### Gene flow and demographic history

To test for present-day and historical gene flow between *R*. *spectrum* populations and to identify population boundaries, we used the R package delimitR [53]. We defined four populations based on potential geographic barriers (rivers, ocean, lowland, and highland) as observed in similar recent studies: the samples from Mt. Cameroon, Kupe, Nlonako, and Manengouba are grouped and labeled continental Cameroon Volcanic Line (CCVL), and the three other populations are Bioko Island, Korup National Park, and Gabon. Using one randomly chosen SNP per ddRAD locus, assuming therefore that our loci are unlinked, we constructed seven folded multidimensional site frequency spectra (mSFS) (S6 Table) using easySFS module [54] implemented in Python v3.9 [55]. In the module, we further restricted our data by downsampling the number of individuals in each population to decrease the frequency of rare sites as suggested by [54].

DelimitR uses a binned multidimensional folded site frequency spectrum (bSFS) [56] and a random-forest machine-learning algorithm to compare speciation models such as no divergence, divergence with and without gene flow, and divergence with secondary contact [53]. A bSFS was used because it stores the observed frequencies of the minor alleles for multiple populations and bins them to avoid inference problems associated with sampling too few segregating sites [56, 57]. DelimitR was chosen over more traditional multi-species coalescent methods because of its ability to readily take historical and current gene flow into account [53, 58]. Demographic histories were simulated using the multi-species coalescent model implemented in fastsimcoal2 [59] under a user-specified guide tree and set of priors on divergence times, population sizes, and migration rates. The random-forest classifier then creates a user-defined number of decision trees from a subset of the prior. Each decision tree compares the empirical bSFS to the SFS of each simulated speciation model and votes for the most likely generating model. The demographic model with the largest number of votes is chosen as the best model. Out-of-bag error rates are used to assess the power of the random-forest classifiers. The posterior probability of the best model is then calculated by regressing against the out-of-the-bag error rates following [60].

Historical demography and gene flow were inferred with two separated sets of analysis. The first set consisted of a demographic analysis for species delimitation and gene flow among all four identified populations (CCVL, Bioko, Korup, and Gabon). The second set consisted of six distinct demographic analyses to test for secondary contact and divergence with gene flow between one population and another (S7 Table).

We simulated 10,000 datasets, using the default parameters for 89 models for the first set and four models for the second set of analyses. Priors for all models were drawn from a uniform distribution for population size: 1,000–1,000,000 haploid individuals (twice the number of estimated diploid individuals). The divergence time: (5, 9), (6, 12), and (9, 13) in millions of generations for the three internal nodes were overlapping and the command myrules was used to specify the order; and migration rates of 0.00000001–0.000005, corresponding to 0.0001–0.05 migrants per generation. Then, we constructed a random-forest classifier using 500 decision trees for 10,000 pseudo-observed data sets for each model. We calculated the out-of-bag error rates, selected the best model given the data and the set of models tested, and approximated the posterior probability of the best model among the 89 simulated models for our overall dataset and the four simulated models.

### Ecological niche modeling

We compiled occurrence data obtained from our field expeditions and records from museum and citizen science platforms (GBIF, iNaturalist, iDigBio), 317 occurrences records were obtained overall (S4 Fig). These occurrence points were then curated by removing duplicate and potentially mislabeled records, then thinned within a range of 10 kilometers. Environmental data were obtained from the WorldClim database v1.4 [61] for 15 of the 19 bioclim variables downloaded at a 2.5-minute resolution. These same 15 variables are used for the Last Glacial Maximum (LGM) of the Pleistocene under three general circulation models: CCSM4, MIROC-ESM, and MPI-ESM-P. Model calibration, creation, projection, and evaluation were conducted using the R package kuenm [62]. Final models were created for each species using the full set of occurrence records and the parameters chosen during model calibration. Models were then thresholded to 5% to create presence-absence maps. Models from each time period were summed to estimate potential LGM and mid-Holocene distributions as well as continuous stability maps [63, 64].

## Results

### Sequencing and RAD data cleaning

We generated new sequences for the mitochondrial *16S* (29 samples, 510 bp) and *ND4* (28 samples, 836 bp) loci, and for the nuclear *RAG1* (20 samples, 1401 bp) marker. The net genetic distance among populations unveiled lower uncorrected *p*-distance among all populations composing the CCVL (Table 1). Our concatenated dataset of 102 samples (S1 Table) consisted of 4,166 bp, including indels. For our ddRAD datasets, the demultiplexing from STACKS produced between 236,072 reads (139,619 loci) and 14,231,248 reads (4,263,311 loci) per sample. The optimized STACKS assembly parameters (m = 3, M = 6, n = 15, r = 0.5 and p = 3) were used to efficiently curate and assemble large numbers of short-read sequences from multiple samples and generate two datasets for subsequent analysis. The first dataset, consisting of one randomly chosen SNP per locus, was used to infer genetic structure; this dataset consisted of 28 samples of *R*. *spectrum* (RADset1). The second dataset, used for phylogenetic analysis, consisted of the 28 samples listed above, plus one sample of *Trioceros cristatus* as the outgroup (RADset 2). RADset1 consisted of 16,354 loci with 54.9% missing data. RADset2 consisted of 16,365 loci with 56.4% missing data.

**Table 1:** Uncorrected *p*-distances among species (bottom matrix) and standard errors (top matrix) in the genus *Rhampholeon* and *R*. *spectrum* species complex populations for two molecular markers a) 16S and b) ND4. The values within each species/population are shown in bold on the diagonal (*p*-distance/standard error). ***na*** denotes not estimated *p*-distances within species/populations counting only one sample.

**a)**	***Rhampholeon***	**1**	**2**	**3**	**4**	**5**	**6**	**7**	**8**	**9**	**10**
**1**	***spectrum* Gab**	**0.0028/0.002**	0.0073	0.0126	0.0126	0.0145	0.0144	0.0139	0.0273	0.0237	0.0269
**2**	***spectrum* Kor**	0.0207	**0.005/0.0023**	0.0107	0.0116	0.0126	0.0125	0.0121	0.0258	0.0236	0.0255
**3**	***spectrum* Bio**	0.0473	0.0362	**0.0008/0.0011**	0.0075	0.0088	0.0088	0.0088	0.0234	0.0226	0.0242
**4**	***spectrum* Cam**	0.0434	0.0362	0.0179	**0.0114/0.0056**	0.0047	0.0048	0.0045	0.0232	0.0230	0.0248
**5**	***spectrum* Man**	0.0499	0.0384	0.0231	0.0085	**0/0**	0.0018	0.0036	0.0249	0.0248	0.0260
**6**	***spectrum* Nlo**	0.0485	0.0370	0.0233	0.0097	0.0015	**0.0014/0.0014**	0.0033	0.0247	0.0247	0.0259
**7**	***spectrum* Kup**	0.0465	0.0366	0.0244	0.0099	0.0056	0.0043	**0/0**	0.0246	0.0244	0.0257
**8**	*spinosus*	0.1121	0.1038	0.0935	0.0911	0.0998	0.0990	0.0994	**0/0**	0.0174	0.0182
**9**	*viridis*	0.0952	0.0916	0.0861	0.0908	0.0974	0.0967	0.0972	0.0636	**0.0142/0.0056**	0.0162
**10**	*temporalis*	0.1068	0.0984	0.0937	0.0958	0.1015	0.1008	0.1013	0.0649	0.0564	**0/0**
**b)**	***Rhampholeon***	**1**	**2**	**3**	**4**	**5**	**6**	**7**	**8**	**9**	**10**
**1**	***spectrum* Gab**	**0.0028/0.0017**	0.0142	0.0189	0.0192	0.0212	0.0219	0.0192	0.0528	0.0408	0.0456
**2**	***spectrum* Kor**	0.0493	**0.0028/0.0015**	0.0191	0.0164	0.0198	0.0208	0.0187	0.0499	0.0413	0.0433
**3**	***spectrum* Bio**	0.0685	0.0690	**0.0018/0.001**	0.0185	0.0207	0.0213	0.0196	0.0559	0.0415	0.0451
**4**	***spectrum* Cam**	0.0674	0.0562	0.0655	**0.0283/0.0096**	0.0096	0.0090	0.0079	0.0483	0.0432	0.0444
**5**	***spectrum* Man**	0.0797	0.0741	0.0773	0.0272	**0.0001/0.0002**	0.0029	0.0073	0.0571	0.0423	0.0444
**6**	***spectrum* Nlo**	0.0819	0.0769	0.0791	0.0247	0.0045	**0.0012/0.0009**	0.0067	0.0569	0.0427	0.0461
**7**	***spectrum* Kup**	0.0692	0.0682	0.0709	0.0192	0.0205	0.0182	**0/0**	0.0548	0.0413	0.0438
**8**	*spinosus*	0.1957	0.1884	0.2111	0.1801	0.2137	0.2123	0.2047	**na**	0.0372	0.0356
**9**	*viridis*	0.1526	0.1557	0.1578	0.1614	0.1613	0.1607	0.1573	0.1468	**na**	0.0309
**10**	*temporalis*	0.1775	0.1683	0.1758	0.1666	0.1721	0.1764	0.1699	0.1405	0.1210	**na**

### Phylogenetic relationships and estimation of the temporal framework for diversification

#### Maximum likelihood and Bayesian phylogenetic inference from the Sanger dataset

**Fig 3:**
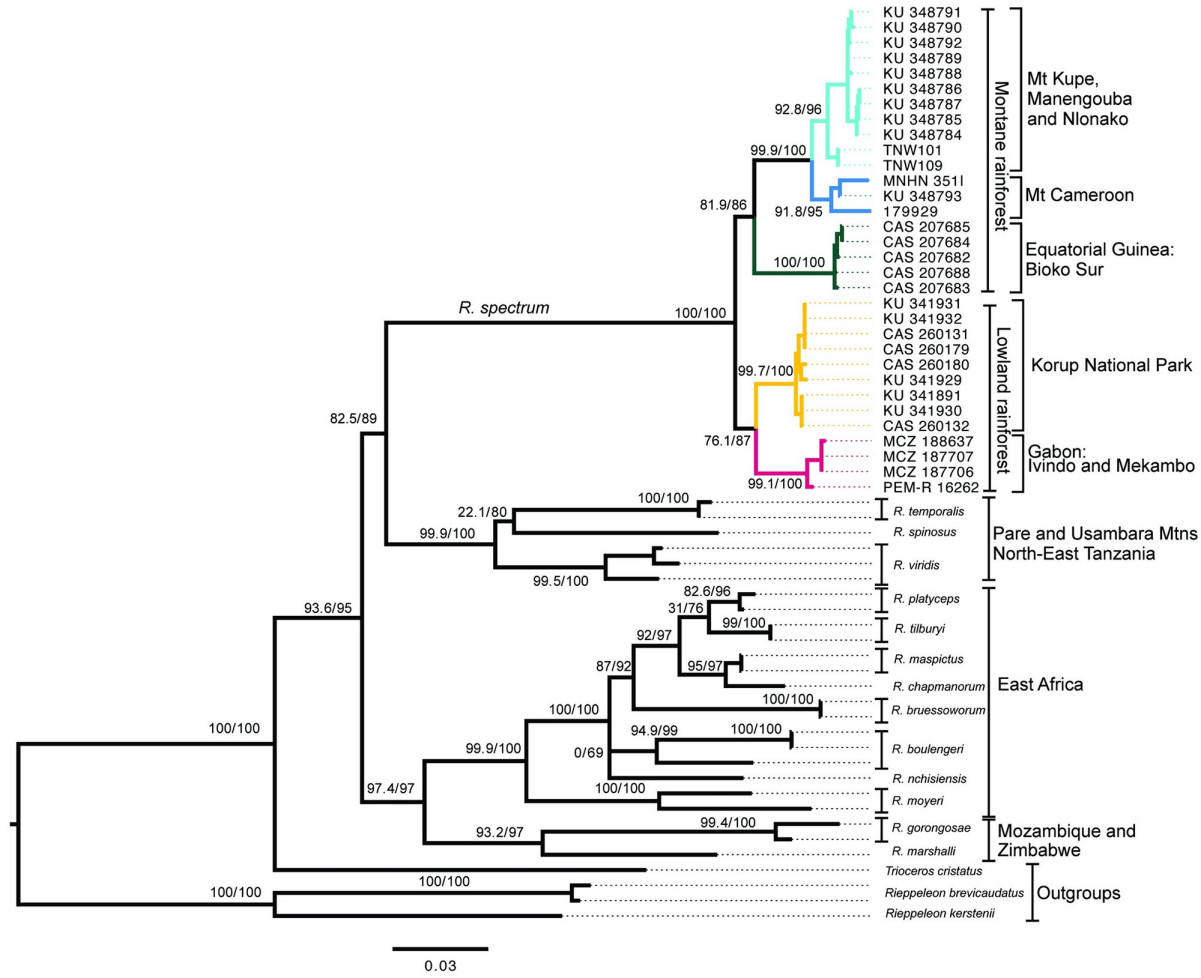
Maximum likelihood phylogenetic tree inferred from *16S*+*ND4*+*RAG1* data set in IQ-TREE. Node values represent SH-aLRT/Ultrafast bootstrap supports in percentage. Branch colors correspond to key in Fig 2.

**Fig 4:**
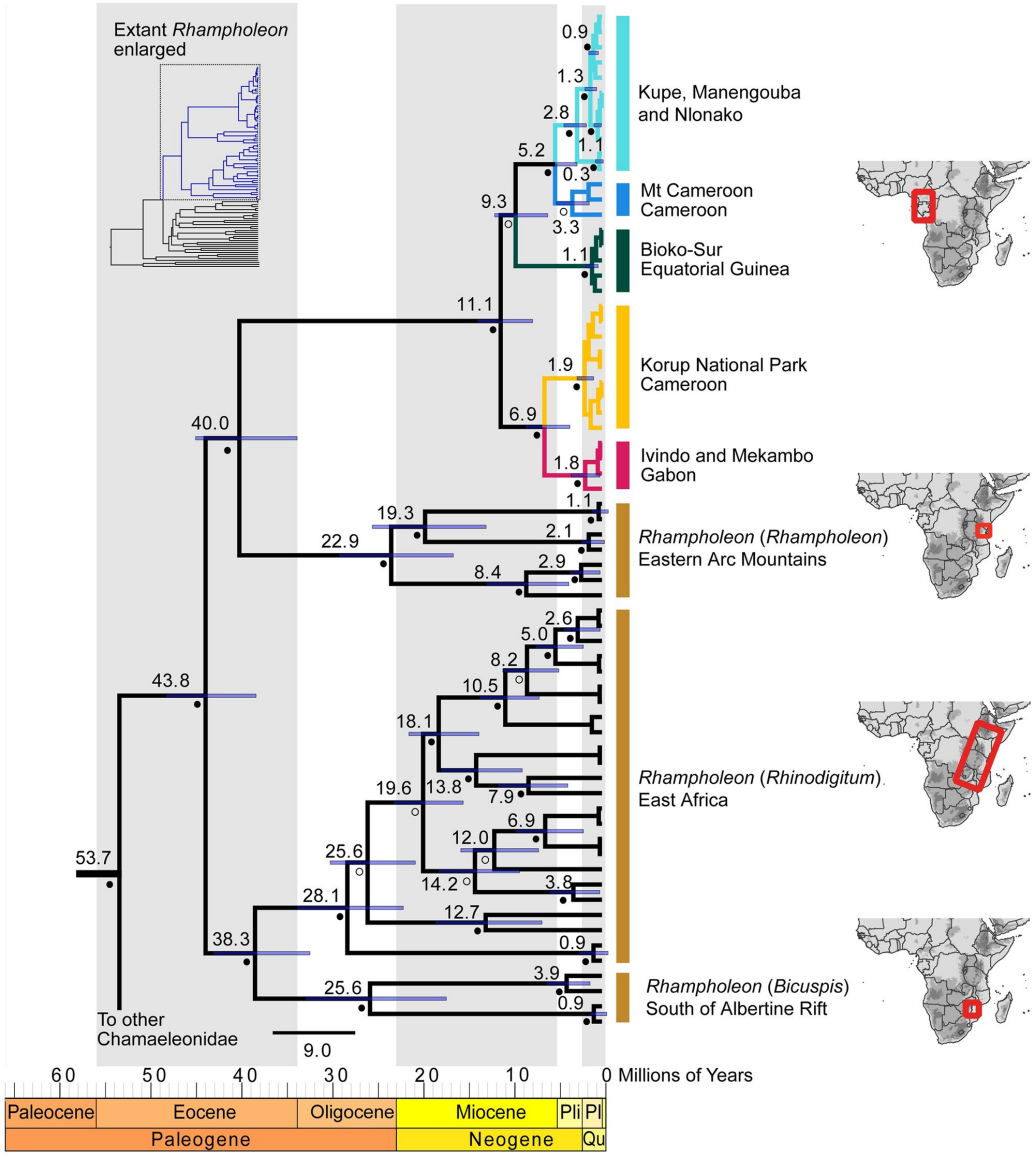
Bayesian chronogram of the Pygmy Chameleon genus *Rhampholeon* inferred from *16S*+*ND4*+*RAG1* data. Nodes with high support (≥ 95%) are denoted by filled circles adjacent to nodes and posterior probabilities (< 95%) are denoted with empty circles. Median ages are provided above nodes and blue bars at nodes represent 95% highest probability densities (HPD). The spatial distribution of populations is presented with the same color scheme in Fig 2. Pli = Pliocene, Pl = Pleistocene, Qu = Quaternary.

The ML phylogenetic tree suggests that *R*. *spectrum* populations from the Lower Guinean Forest are sister to the montane endemic chameleons (*R*. *spinosus*, *R*. *temporalis*, and *R*. *viridis*) from the Eastern Arc Mountains (Pare and Usambara Mts) in Tanzania (Fig 3). *Rhampholeon spectrum* is composed of one lowland clade and one montane clade. The lowland clade is composed of two populations; samples from Mekambo and Ivindo comprise the Gabon population, whereas the Korup National Park samples (Cameroon) form the Korup population. The montane clade is composed of three populations. Samples from Mt. Biao on Bioko Island form the Bioko population, samples from Mt. Cameroon comprise the second montane population, and samples from the geographically proximate Kupe, Nlonako, and Manengouba mountains form the third population (Fig 2). Hence, our concatenated dataset recovered five distinct lineages within *R*. *spectrum* (Figs 3 and 4) supported by at least one phylogenetic inference method. The sample MNHN 351I from Mapanja (Mt Cameroon population) was collected close to the type locality of *R*. *spectrum*, with Bonjongo being located less than 3 km from Mapanja. Therefore, the Mt. Cameroon population can be considered here as our topotypic group.

The split between the *R*. *spectrum* species complex from West-Central Africa and the montane endemic clade from the Eastern Arc Mountains of Tanzania occurred in the Eocene ~40 Mya. Within *R*. *spectrum*, the divergence between the lowland and montane clades occurred around the mid-Miocene ~11.1 Mya. This event was followed by the divergence of the Bioko population from the continental CVL ~9.3 Mya. The initial divergence between the two lowland populations (Gabon and Korup) is estimated to have occurred in the late Miocene (~6.9 Mya). The earliest divergence within the CCVL lineage happened during the Miocene-Pliocene transition (~5.2 Mya) and produced the Mt. Cameroon population and the Kupe, Nlonako, and Manengouba populations. Overall, all five newly discovered lineages within *R*. *spectrum* arose between the middle- and late-Miocene (Fig 4).

#### Species tree inferred from ddRAD datasets

The loci obtained from the RADset2 were used to infer a maximum likelihood tree using IQ-TREE (S2 Fig) and a species tree using SVDQuartets (S3 Fig). High bootstrap supports (>90) were observed for most nodes in the SVDQuartets species tree, except for the nodes Korup+Gabon (60) and Gabon (66). The monophyly of the montane clade (Bioko + CCVL) is strongly supported by both trees. The species tree, the Bayesian tree, and the maximum likelihood tree were all topologically similar among our five populations. The monophyly of CCVL is strongly supported in all four phylogenetic trees inferred. The only observed topological differences among estimates of phylogeny conducted here was the RADset2 maximum likelihood tree, which suggests the monophyly of Korup+Bioko+CCVL population, which is sister to the Gabon population (S2 Fig).

### Population genetic structure

**Fig 5:**
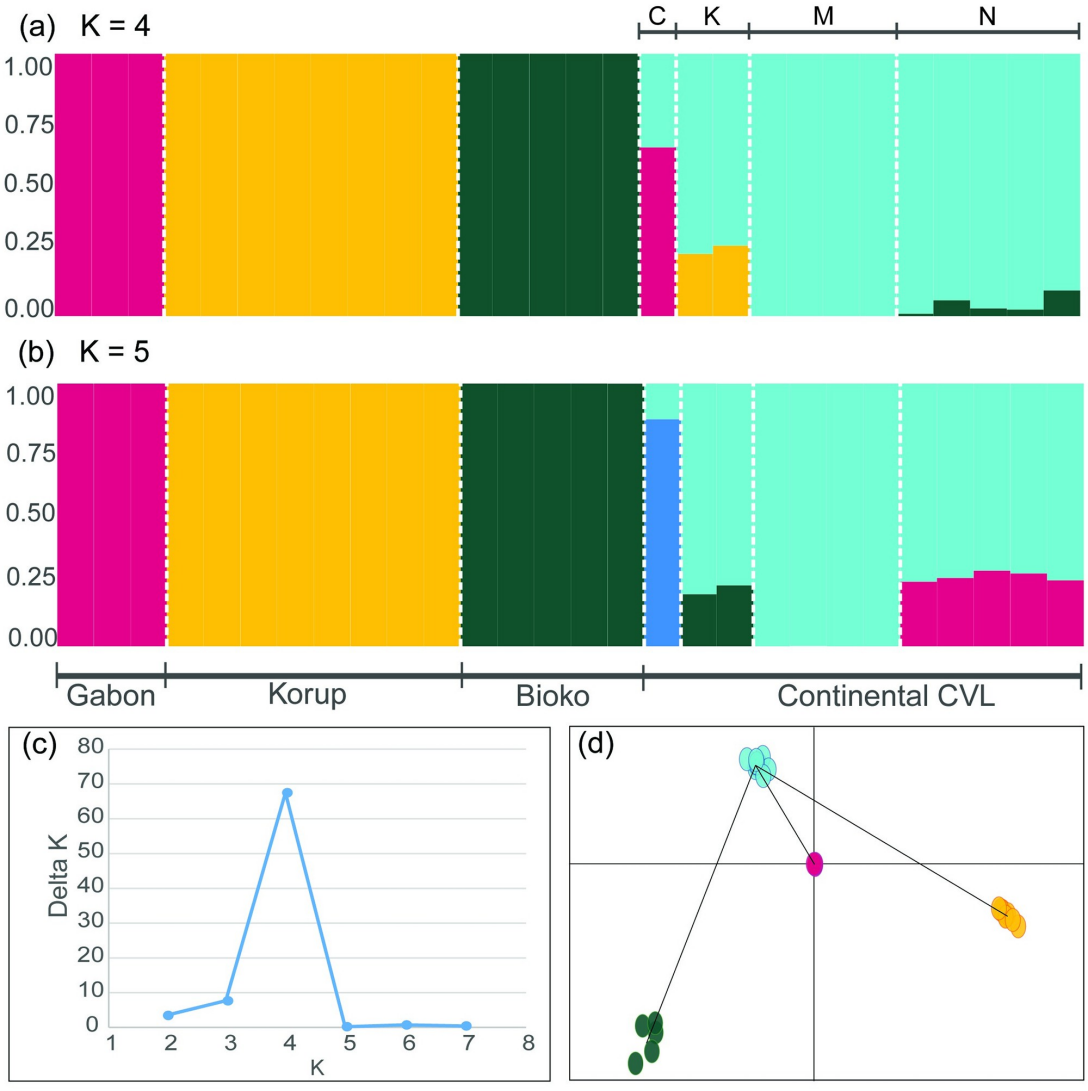
Bayesian cluster analysis using the STRUCTURE program for 28 *Rhampholeon spectrum*. (a) plot for K = 4, (b) plot for K = 5, (c) delta K from the structure analysis was calculated according to the method of Evanno from POPHELPER, and (d) a discriminant analysis of principal components using the program Adegenet. In (a) and (b), each bar corresponds to one sample of *R*. *spectrum*. C = Mt. Cameroon, K = Mt. Kupe, M = Mt. Manengouba, N = Mt. Nlonako, K = Number of populations, CVL = Cameroon Volcanic Line. The color scheme matches the sampling localities depicted in Fig 2.

The Bayesian clustering analysis is based on 16,166 randomly selected SNPs that were sampled across 28 samples of *R*. *spectrum* (Fig 5). The optimal number of populations is K = 4 (Fig 5a). The high value of Delta K obtained (Fig 5c), and the plotting of discriminant analysis of principal components support this result further. The four clusters corresponded to the populations of CCVL, Bioko, Korup, and Gabon (Fig 5a and 5d). When plotting the clusters for K = 5. The single RADseq sample from Mt. Cameroon made up the fourth cluster, and the Kupe, Nlonako, and Manengouba populations comprised the fifth cluster (Fig 5b). According to the bar plot obtained from STRUCTURE (Fig 5a), only the CCVL cluster shows evidence of admixture.

### Gene flow and demographic model selection

We performed demographic model selection with two separate sets of analyses. The first set consisted of a demographic analysis for species delimitation and gene flow among all four identified populations identified with Structure (CCVL, Bioko, Korup, and Gabon). The second set consisted of six distinct pairwise demographic analyses to test for secondary contact and divergence with gene flow between pairs of populations (S5 Table).

**Fig 6:**
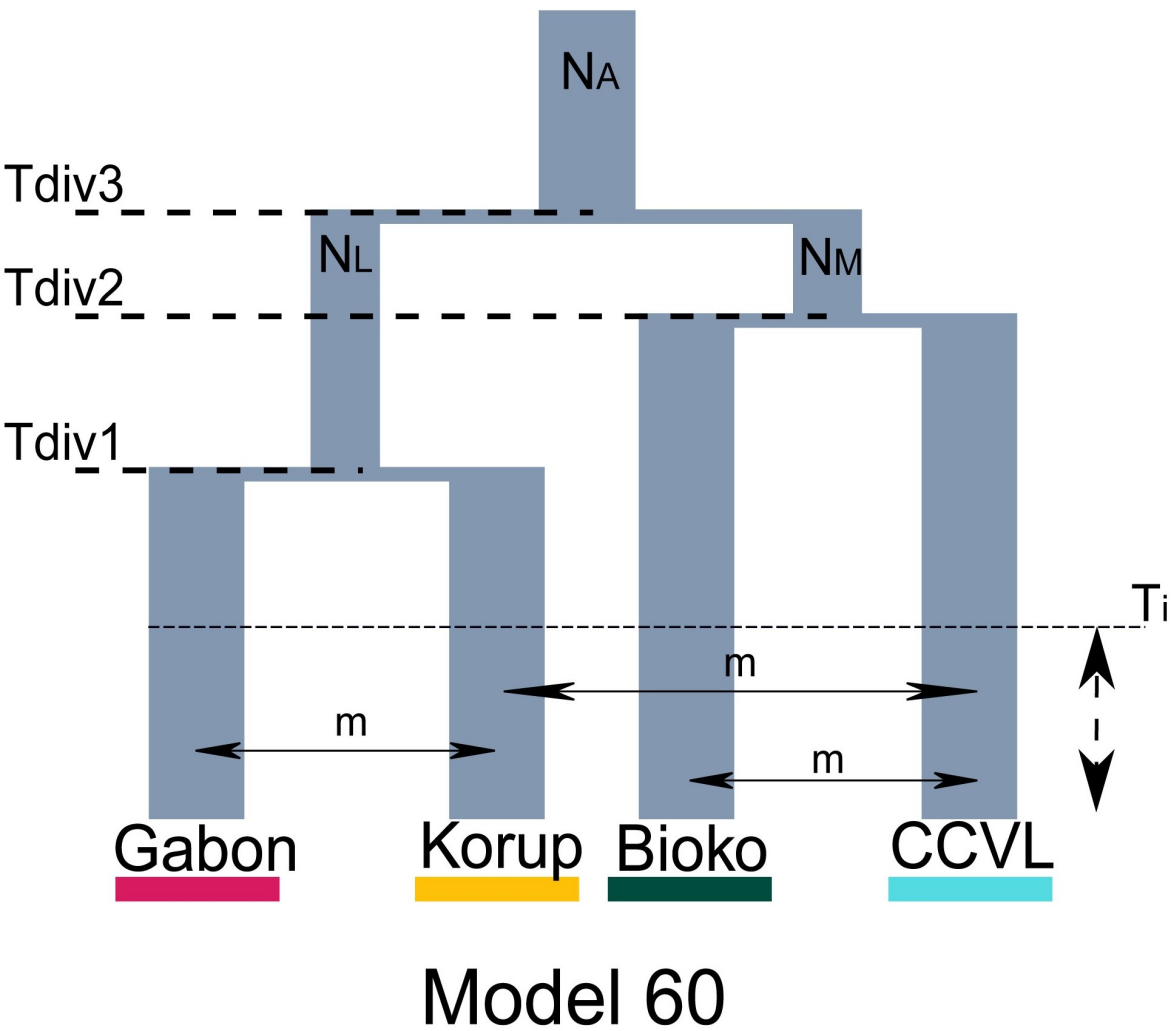
Results of demographic model comparisons among all four clades. The best migration and species delimitation model generated from multidimensional site frequency spectrum inferred with FastSimcoal2 implemented in DelimitR. N_A_: Ancestral population, N_L_ = Lowland population, N_M_ = montane population, T_div_ = Divergence time, T_i_ = Time since isolation, m = migration.

For the first set of demographic analyses, the multispecies site frequency spectrum (mSFS) was constructed (after down sampling) using five samples for CCVL, three from Bioko, four from Korup, and two from Gabon. Our mSFS was built from 1,220 unlinked SNPs sequenced across all four populations. DelimitR produced 89 models to test for species-level divergences with gene flow and with secondary contact. Sixty-six of the 89 models support four distinct populations hypothesis, and they share 428 of the 500 votes (S4 Table). Model 60 (Fig 6) was selected with 27 votes as the best-supported evolutionary scenario, with an out-of-bag prior error rate of 29% and a posterior probability of 0.48. This model supports the diversification of *R*. *spectrum* into four distinct populations as identified above, followed by secondary contact between Gabon and Korup populations, between CCVL and Korup, and between Bioko and CCVL populations (Fig 6). The confusion matrices and the overall numbers of votes per models are provided in S4 and S5 Tables.

**Fig 7:**
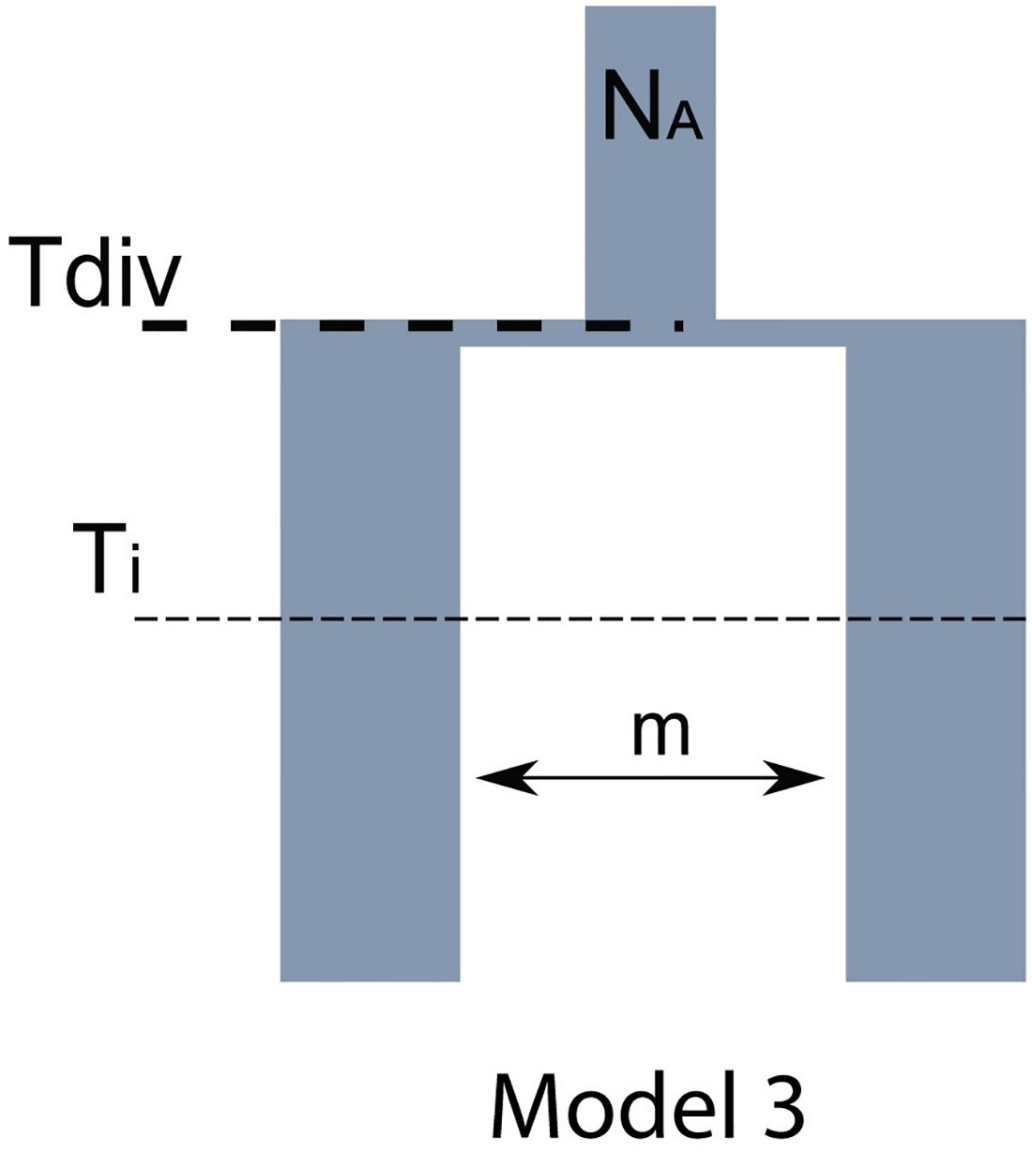
Results of demographic model comparisons between all populations. The best migration and species delimitation model generated from multidimensional site frequency spectrum inferred with FastSimcoal2 implemented in DelimitR. N_A_: Ancestral population, T_div_ = Divergence time, T_i_ = Time since isolation, m = migration.

For the second set of analysis, six mSFS were built using four samples from Bioko, five from Korup, six from CCVL, and two from Gabon. The numbers of unlinked SNPs used to build each of the six interactions are listed in S5 Table. Four models were created: (1) no divergence (a single population), (2) divergence without gene flow, (3) divergence with secondary contact, and (4) divergence with gene flow. For all six tests performed in delimitR, divergence with secondary contact was selected as the best model supported (Fig 7), with a posterior probability ranging 0.96–1.00, votes ranging 359–500 out of 500 random forest classifiers, and an out-of-bag prior error ranging 8.07–16.00% (S7 Table). The confusion matrix and the overall number of votes per models are in S7 and S8 Tables.

### Ecological niche modeling

We used ecological niche modeling to explore changes in the potential geographic distribution over the last 20,000 years (i.e., between the last glacial maximum, Mid-Holocene, and the present time). First, despite the presence of suitable conditions in the Upper Guinea region and on the islands of São Tomé and Principe, this species has not been recorded from these areas. Most likely, these species were never able to cross the Niger Delta or the intervening parts of the Gulf of Guinea (S4 Fig).

During the LGM, the area highly suitable for the species was quite small and associated with areas identified as putative Pleistocene refugia by Maley [65]. It is important to note that a habitat connection between the continent and the island of Bioko, inferred to have existed under LGM conditions, and which exposed a land bridge and suitable conditions for this species (S5B Fig). Model transfers to Mid-Holocene conditions show a potential range expansion toward the East and the presence of suitable habitat in the Congo Forest (S5A Fig); LGM suitability patterns are similar to the mid-Holocene. However, the range of the present-day suitability appears to be slightly smaller than current distribution polygon obtained from present day species occurrences from direct observations and museum database (S4 Fig).

**Fig 8:**
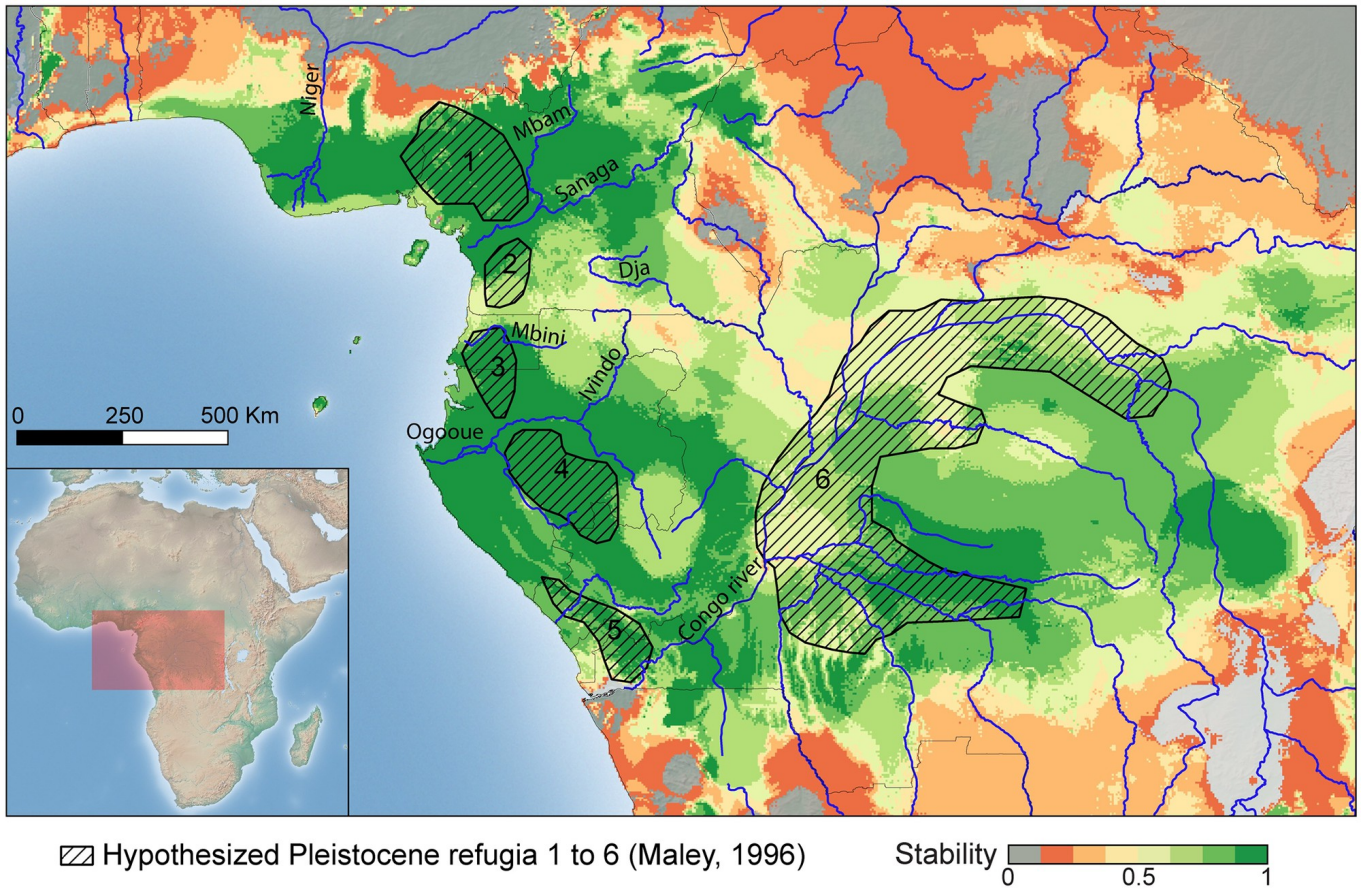
Stability map representing regions of persistent suitable habitat for *Rhampholeon spectrum* species complex across LGM and current climate regimes. The green color represents the highest habitat stability inferred. 1: Cameroon Volcanic Line; 2: Ngovayang and surrounding massifs; 3: Monts de Cristal; 4: Monts Doudou; 5: Massif du Chaillu; and 6: Congo River.

Our species distributional modeling predicts three main high-stability habitats: north of the Sanaga River, south of the Mbini River, and east of the Congo River (Fig 8). The region east of the Congo River represents the area with the fewest occurrence records of *R*. *spectrum*. All high-stability surfaces encompass part or the entirety of previously proposed Pleistocene refugia [65] (Fig 8). The stability regions west of the Congo River contain topographical variation with high elevation and low elevation regions.

## Discussion

In this study, we characterized the temporal and geographical framework for diversification in *Rhampholeon* of West-Central Africa using phylogeographic analyses and historical demographic model selection techniques in order to test predictions derived from alternate hypotheses of mechanisms of diversification and possible speciation. To relate diversification to the variable topography and environments of West-Central Africa, we considered historical isolation-by-barriers such as elevational relief (mountains) and major rivers (Sanaga and Ogooué), hypothetical montane and lowland forest refugia, climatic oscillation, and temporary oceanic land bridges in the genetic structure of our extant populations.

Our results suggest that these factors may have jointly combined in complex ways to influence diversification, ultimately giving rise to geographically structured genetic variation across the variable and disjunct geographical distribution of *R*. *spectrum*. Despite the lack of genetic samples from southern and eastern Cameroon, continental Equatorial Guinea, southern Gabon, and the Republic of Congo, we found corroboration with past geographic events and climatic fluctuation, which appears to explain the surprising and previously undocumented concordance between genetic structure and geographical features in this anomalously distributed Pygmy Chameleon. We interpret geographically based genetic variation elucidated here as clearly associated with (1) the Last Glacial Maximum refugia, (2) a temporary land bridge that connected Bioko Island and the African continent, (3) the uplift of the Cameroon Volcanic Line (lowland vs. highlands) and (4) the major rivers of the Lower Guinean Forest (Congo, Sanaga and Ogooué/Ivindo Rivers). Below, we summarize these major findings and discuss the implications, representing research and conservation priorities for future work on diversification of vertebrates in the central African tropics.

### Systematics

Our phylogenetic analyses of the genus *Rhampholeon* recovered the same topology reported in previous studies [1, 3–5, 36, 66]. Despite the geographical intermediacy of some members of the subgenus *Rhinodigitum* (especially *R*. *boulengeri* found in eastern Congo) between *R*. *spectrum* and its sister clade from Tanzania, these two clades (*R*. *spectrum* group and the *Rhinodigitum* subgenus) are polyphyletic [1, 5]. Similar disjunct east-west distributional patterns in Chamaeleonidae have been observed in the genus *Chamaeleo* [23, 36].

Our novel phylogenomic analyses of *R*. *spectrum* reveals the previously unknown existence of five genetically divergent, geographically circumscribed lineages/populations, nested within two major ecologically defined clades: a montane clade, and a lowland clade (Fig 3). The two lowland populations were sampled from forested sites in northeastern Gabon and Korup National Park, at the southwestern border between Cameroon and Nigeria. Separated by ≥ 500 kilometers (Fig 2), the lowland populations form a monophyletic group, sister to a clade composed of montane populations, sampled from sites along the Cameroon Volcanic Line (≥ 700 m). The Cameroon Volcanic Line is known to be a hotspot for endemism and speciation in continental Africa [67–69].

Systematic studies of East African members of the genus *Rhampholeon* [1,6] and other chameleons [70, 71] have resulted in recently described species, all of which relied, in part, on phylogenetic support and levels of genetic divergence (uncorrected *p*-distances), similar to those reported here in Table 1, as justification for their formal taxonomic recognition. Based on the geographic distribution of genetic diversity we recovered, our results support a range of possibilities, likely for at least two potentially cryptic species within the *R*. *spectrum* complex. Thus, this study sets the stage for a comprehensive taxonomic investigation of the species, based on robust statistical species delimitation analyses of genomic data, consideration of name-bearing type specimens, and recently accumulated specimens and their associated data from West-Central Africa.

### *Rhampholeon spectrum* paleo-diversification through time

The West-Central African *Rhampholeon spectrum* split from the South Pare and Usambara Mountains group of East Africa (*R*. *spinosus*, *R*. *temporalis*, and *R*. *viridis*) during the late Eocene around 40 Mya. This period corresponded to the break-up of West-Central and East African forests [72, 73] and subsequent diversification within the *R*. *spectrum* clade likely took place in the Miocene (Fig 4). The mid-Miocene diversification between the lowland and montane forest clades corresponds to the uplift of the Central African Atlantic Swell, a low mountain range (maximum elevation 1200 m) stretching from southern Cameroon to southern Republic of Congo [74]. Similar diversification patterns were observed in puddle frogs of the Cameroon Volcanic Line [69, 75].

The period from the end of the Miocene to the Pleistocene corresponds to an acceleration of lineage accumulation in *R*. *spectrum*. The cycles of forest expansion and contraction during the Pliocene-Pleistocene may have increased allopatric speciation rates for forest-adapted lineages [73]. Initial divergence within the Bioko Island population, around 9 Mya (Fig 3), appears to be older than the age of the island itself (approximately 1.33 Mya) [76–78]. Our corresponding branch is poorly supported in our Sanger phylogenies (Figs 3 and 4) but strongly supported in our genomic ddRAD topology (S2 and S3 Figs). Two hypotheses could explain the inferred age of this population. First, Bioko population could represent a relictual distribution: a lineage formerly more widespread, which may have been restricted to coastal regions prior to the uplift of Bioko Island, and subsequently leading to the colonization of Bioko, followed by the extinction of the continental population [79–81]. Second, its estimated age could be an artifactual result of outgroup calibration [82, 83].

Many amphibian and reptile species from the Lower Guinean Forest have a sister species found in Upper Guinean Forest [21, 84], but the genus *Rhampholeon* seems not to have crossed the Niger Delta. It is likely that the Niger River and the uplift of the Cameroon Volcanic Line during the Eocene created dispersal barriers for this species, which has its western distributional limit at the Cross River in Nigeria (S4 Fig). We found evidence that paleoendemic lineages persisted in montane forest refugia since the Eocene [85].

### Demographic inference and ecological niche modeling

Demographic modeling supports the four distinct populations of *Rhampholeon spectrum* (CCVL, Bioko, Korup, and Gabon) and identified divergence with secondary contact as the most likely demographic scenario. This result could be explained by temperature oscillations and habitat contraction during the Pliocene-Pleistocene [73]. Taking in consideration the hypothesized Pleistocene montane and lowland forest refugia, the pairwise demographic model tests between all four populations found support for a model of divergence with secondary contact as well. This outcome is supported by the potential role of Mid- and Late Pleistocene climatic oscillations [86, 87] and lowland forest refugia in facilitating gene flow between divergent lineages/species. The shallow channel between Bioko Island and continental Africa is only 60 m deep; on average, whereas global sea levels dropped ≥ 100 m during the Last Glacial Maximum (S5B Fig). Similar sea level changes have occurred several times during the Ice Age [88–91]. Together, these observations support the hypothesis of secondary contact between *R*. *spectrum* on the island of Bioko and the continental populations.

Our species distribution model for *R*. *spectrum*, over the last 22,000 years, encompasses several areas of stability at high elevation. Previous studies have asserted that topologically complex mountains with pronounced geodiversity, may be correlated with high levels of biodiversity [69, 92–94]. *Rhampholeon spectrum* is a leaf-litter dwelling chameleon found exclusively in the rainforest of West-Central Africa. We identified three major population contractions, and their geographic locations overlap with hypothesized refugia. The suitable habitat identified in the heart of the Congo could suggest that a paucity of genetic material from this region may be to blame for our inferred lack of Eocene–Miocene diversification in our time-calibrated phylogenetic estimate.

## Conclusions

Two distinct mechanisms, vicariance on the African continent and dispersal via ephemeral land bridges toward the continental island of Bioko, are the main explanations for *R*. *spectrum*’s contemporary phylogeographic patterns. Forest fragmentation-induced vicariance within *Rhampholeon* apparently initiated during the mid-Eocene with the subdivision of sub-Saharan rainforest into small patches during the Paleocene-Eocene. Within the West-Central African *R*. *spectrum*, the diversification into two main lineages during the mid-Miocene corresponds with the uplift of the Cameroon Volcanic Line [95]. These diversification events resulted in the appearance of one ancestral lowland lineage and one ancestral montane lineage, and we found evidence to support the presence of two to five distinct species within our data set. We successfully inferred the putative mechanisms of diversification north of the Sanaga River and provided but more genetic sampling will be needed to develop a full picture of the genetic structure of *R*. *spectrum* in South-East Cameroon, the Republic of Congo, and farther west. In a general sense, these findings highlight the importance of combining genomically informed demographic model selection, dated molecular phylogenies, and distributional stability mapping to draw inferences about the mechanisms that contribute to present-day patterns of the distribution of biological diversity.

Our work represents one of a few recently emerging studies considering alternate processes of diversification and potentially speciation in the Lower Guinean Forest and adjacent islands in the Gulf of Guinea [12, 20, 21, 96]. We highlight newly-elucidated, geographically-based genomic variation, across the range of an endemic, previously unstudied forest-associated vertebrate—all of which enables a comprehensive understanding (and conservation assessment) of the temporal and evolutionary context of putative speciation in a unique forest vertebrate from a celebrated biodiversity hotspot. A taxonomic follow-up study on this species complex using morphology, ecology, and characterizations of gene flow as a final “validation” step for statistical species delimitation could be helpful to determine if the genetic lineages observed in this study’s “discovery” stage analyses might provide actual statistically robust support for actual separate species recognition.

## Supporting information

S1 Fig Bayesian chronogram of the Pygmy Chameleon genus *Rhampholeon*. Numbers near nodes denote median value of node age in millions of years. Letters indicate the nodes used for calibration (S3 Table). Quat = Quaternary.
(TIF)

S2 Fig Maximum likelihood tree estimated from full ddRADseq data set in IQTree. Numerical node support values represent percentages of 100,000 ultrafast bootstrap replicates. Branch lengths are proportional to expected substitutions per site. * Denotes nodes with UFbootsrap >95%.
(TIF)

S3 Fig Species tree estimated from full ddRADseq data sets in PAUP* SVDQuartets. Numerical node support values represent percentages of 500 non-parametric bootstrap replicates.
(TIF)

S4 Fig Locality points used for ecological niche modeling and DNA analysis. (TIF)

S5 Fig Past and present-day distribution models. (A) suitable habitat for *R*. *spectrum* during the mid-Holocene, (B) suitable habitat during the last glacial maximum (LGM), (C) Stability map representing suitable habitat for *Rhampholeon spectrum* persistent across LGM and current climate regimes. In (A) and (B), the shades of green represent agreement between global climate models (GCMs) with the darkest green indicating agreement between all three GCMs and the lightest green indicating support from only one GCM. In (C) Dark green represents the highest habitat stability inferred.
(TIF)

S1 Table GenBank accession numbers (*16S*, *ND4*, *RAG1*) for chameleon species used in this study. N/A = data, specimen, or information not available.
(DOCX)

S2 Table Primers used for sequencing mitochondrial and nuclear genes. (DOCX)

S3 Table Divergence date priors for primary (fossil) and secondary calibrations. Node letters correspond to those in Bayesian tree in supplemental S1 Fig. Posterior ages, in millions of years ago (Mya), are presented as median values and 95% confidence intervals.
(DOCX)

S4 Table Votes out of 500 random forest classifiers for each of the 89 competing demographic models inferred in delimitR. (DOCX)

S5 Table Number of putatively unlinked SNPs used to produce the mSFS between populations for the six pairwise models used to test demographic scenario with ddRAD dataset 2. (DOCX)

S6 Table Confusion matrices from delimitR analyses. Values along the diagonal indicate the number out of 10,000 simulated data sets that were correctly classified by the random forest classifiers. Model 1: no divergence, model 2: divergence without gene flow, model 3: divergence with secondary contact, and model 4: divergence with gene flow.
(DOCX)

S7 Table Votes out of 500 random forest classifiers for each competing demographic model in delimitR. Model 1: no divergence, model 2: divergence without gene flow, model 3: divergence with secondary contact, and model 4: divergence with gene flow.
(DOCX)

S8 Table DelimitR results for the pairwise interaction models. (DOCX)

S1 File Modified ddRADseq protocol. (DOCX)

S2 File (XLSX)
