# Supplementary figures and images for: Diversification and historical demography of Rhampholeon spectrum in West-Central Africa
Source: PLoS One. 2022 Dec 16;17(12):e0277107. doi: 10.1371/journal.pone.0277107 (PMC9757597; doi:10.1371/journal.pone.0277107)

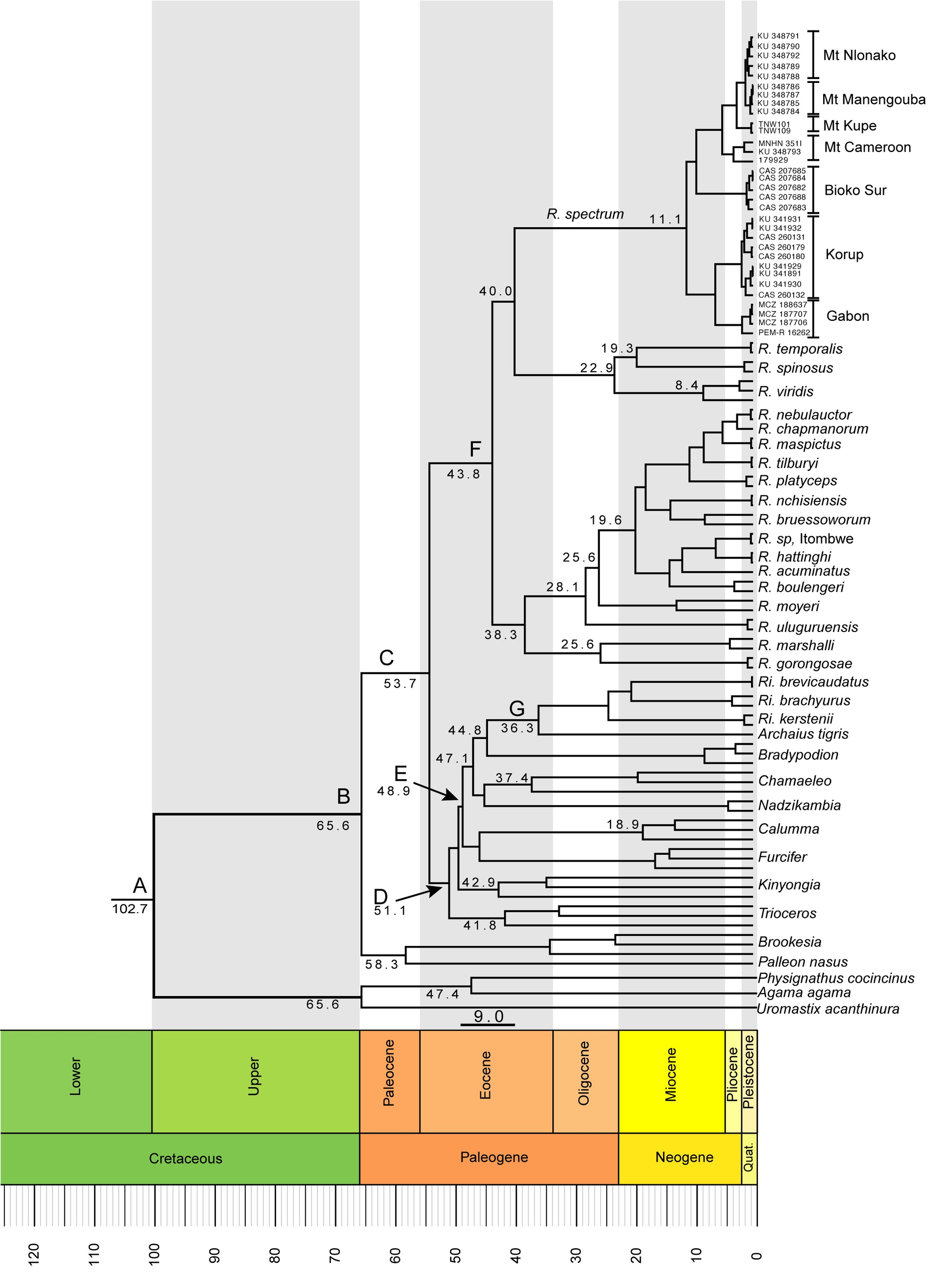

Supplement: S1 Fig — Numbers near nodes denote median value of node age in millions of years. Letters indicate the nodes used for calibration (S3 Table). Quat = Quaternary. (TIF) [file pone.0277107.s001.tif]

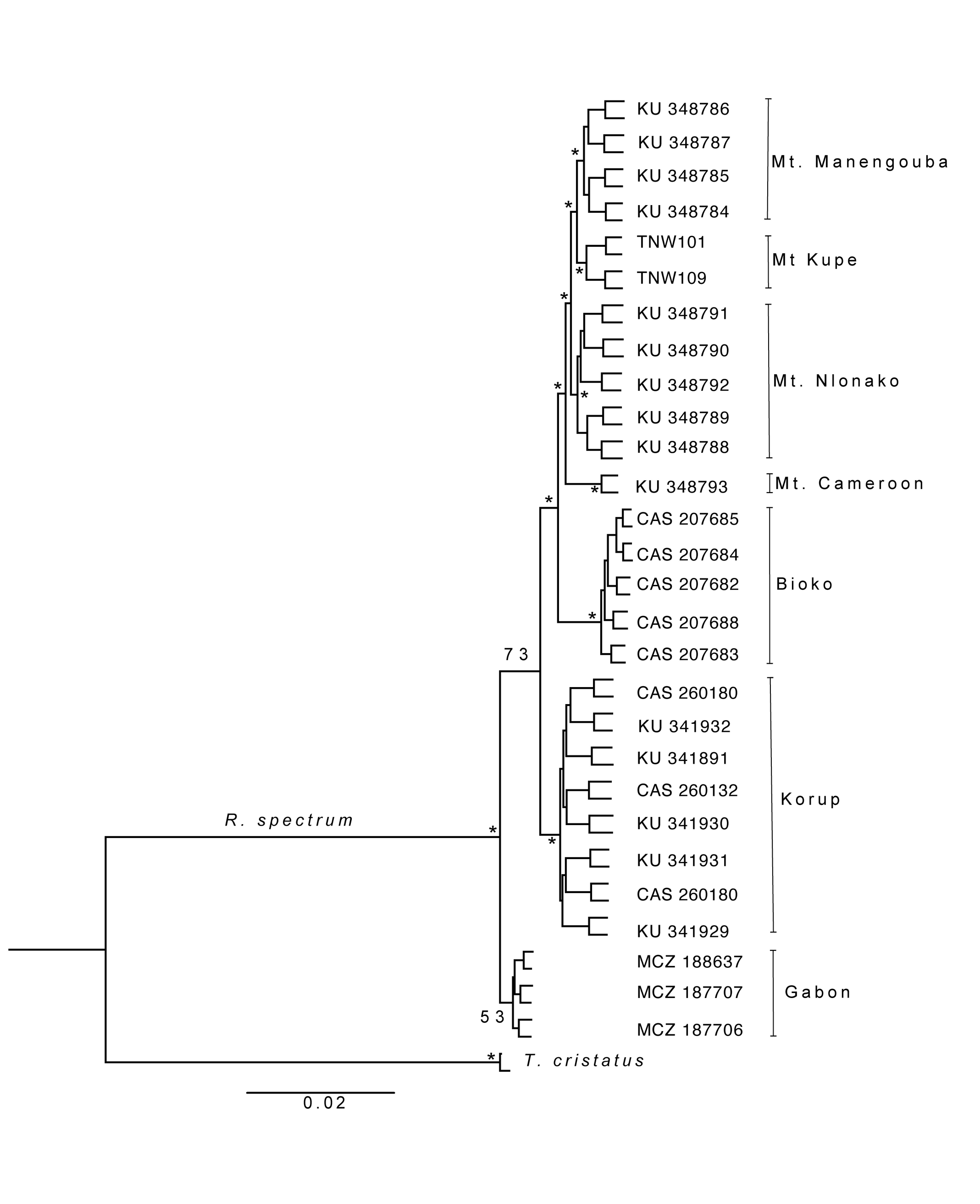

Supplement: S2 Fig — Numerical node support values represent percentages of 100,000 ultrafast bootstrap replicates. Branch lengths are proportional to expected substitutions per site. * Denotes nodes with UFbootsrap >95%. (TIF) [file pone.0277107.s002.tif]

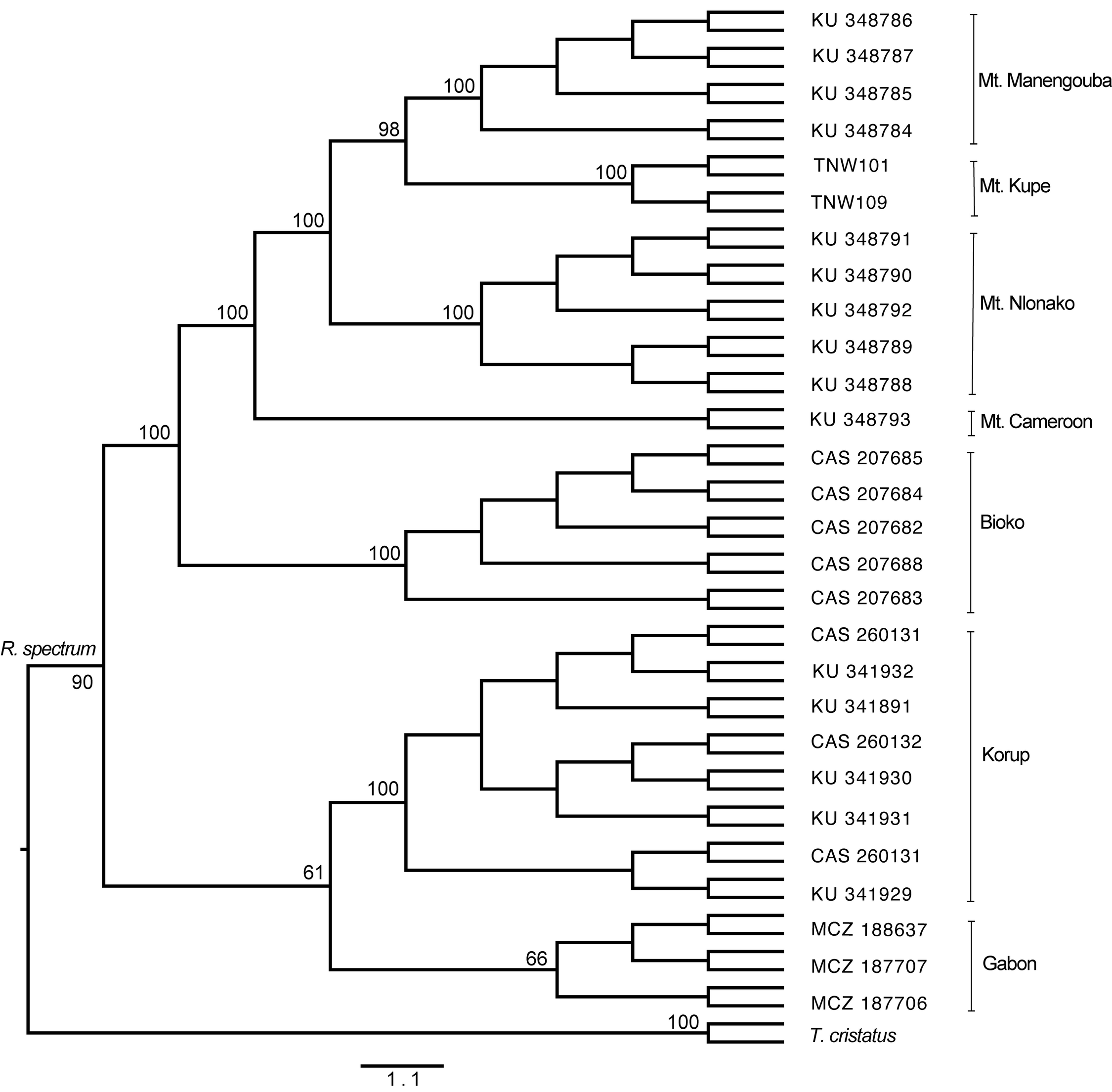

Supplement: S3 Fig — Numerical node support values represent percentages of 500 non-parametric bootstrap replicates. (TIF) [file pone.0277107.s003.tif]

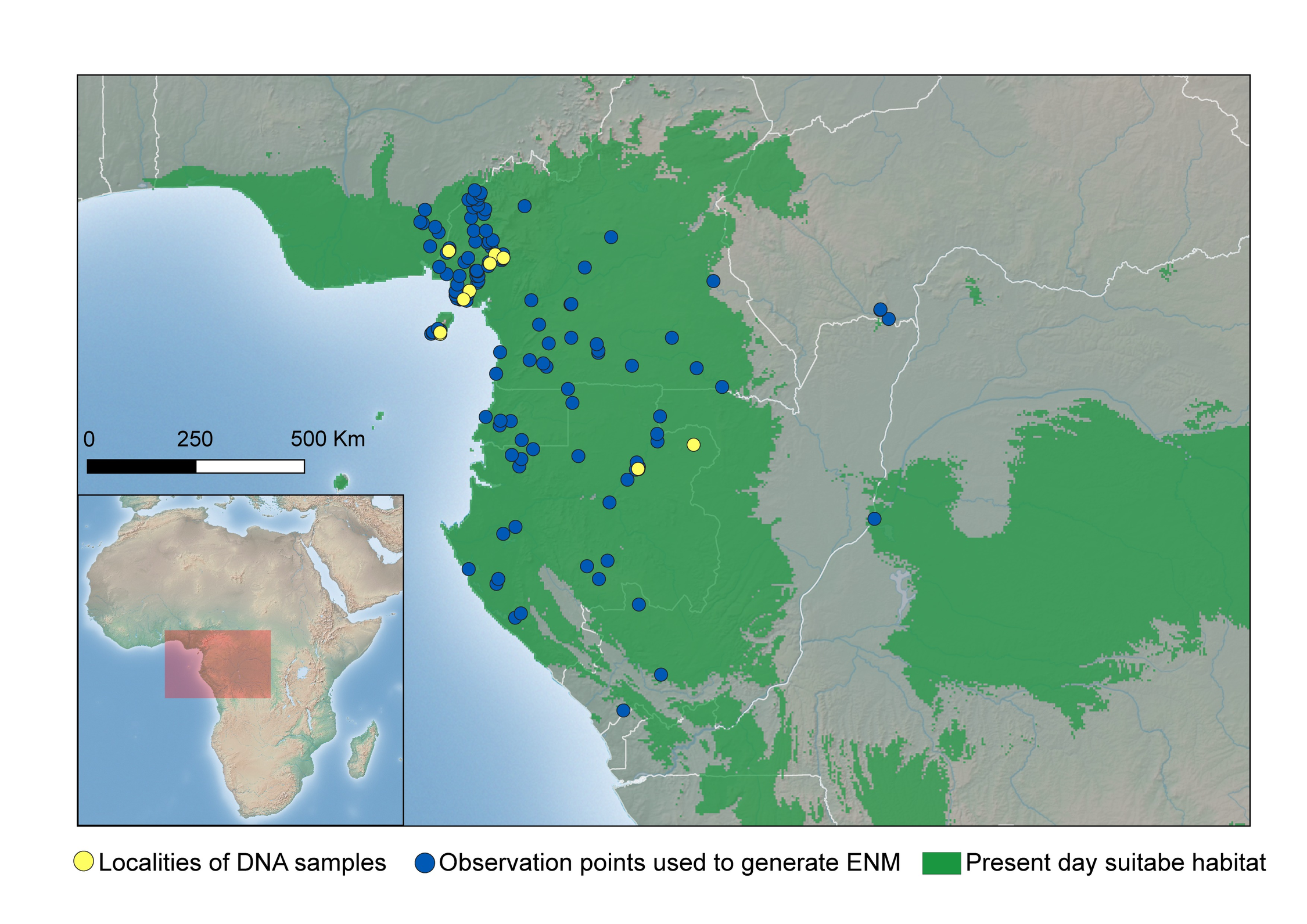

Supplement: S4 Fig — (TIF) [file pone.0277107.s004.tif]

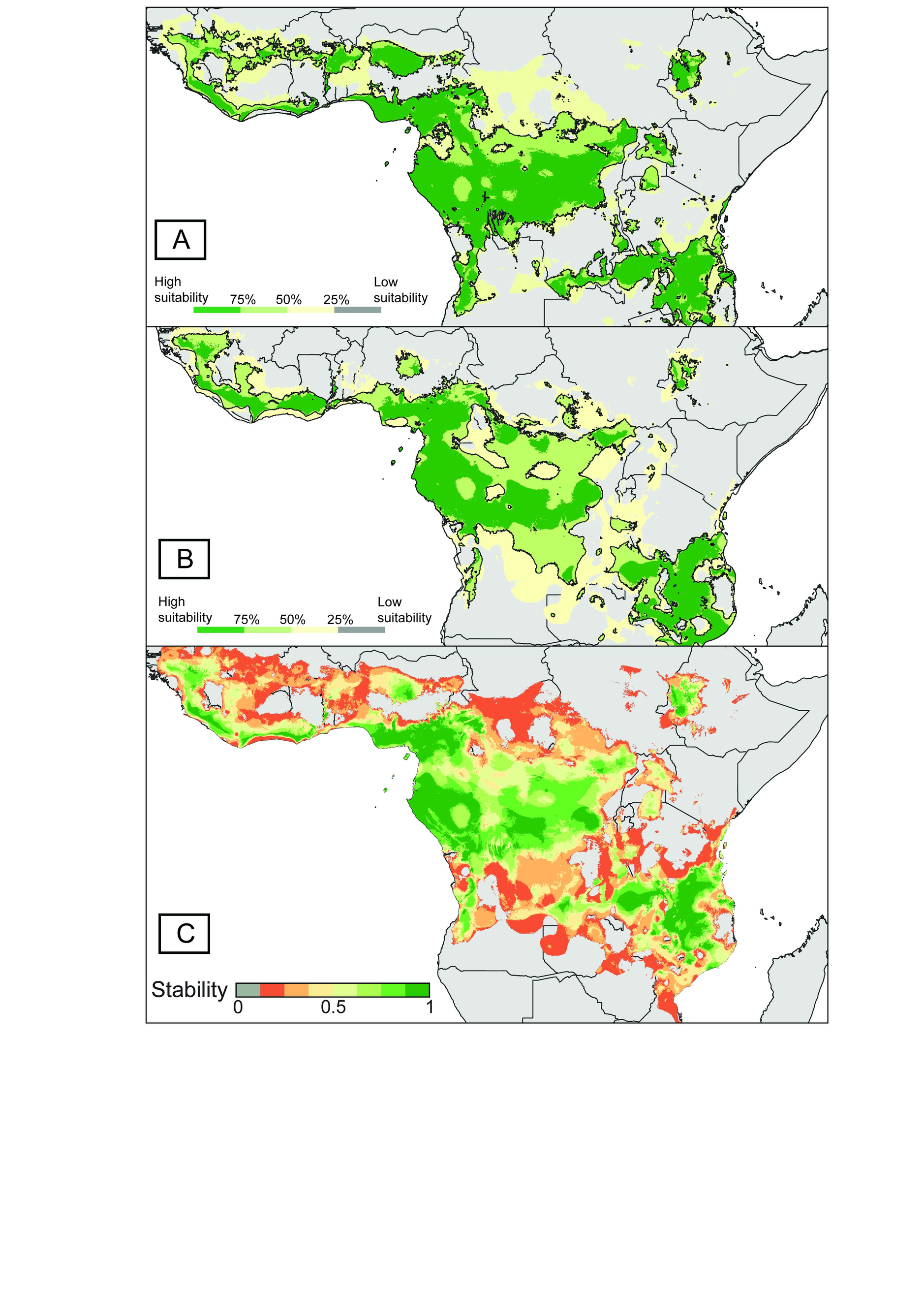

Supplement: S5 Fig — (A) suitable habitat for R. spectrum during the mid-Holocene, (B) suitable habitat during the last glacial maximum (LGM), (C) Stability map representing suitable habitat for Rhampholeon spectrum persistent across LGM and current climate regimes. In (A) and (B), the shades of green represent agreement between global climate models (GCMs) with the darkest green indicating agreement between all three GCMs and the lightest green indicating support from only one GCM. In (C) Dark green represents the highest habitat stability inferred. (TIF) [file pone.0277107.s005.tif]
