# Supplementary material for: Diversification and historical demography of Rhampholeon spectrum in West-Central Africa
Source: PLoS One. 2022 Dec 16;17(12):e0277107. doi: 10.1371/journal.pone.0277107 (PMC9757597; doi:10.1371/journal.pone.0277107)
Supplement: S1 Table — N/A = data, specimen, or information not available. (DOCX) [file pone.0277107.s006.docx]

**S1 Table.** GenBank accession numbers (*16S*, *ND4*, *RAG1*) for chameleon species used in this study. N/A = data, specimen, or information not available.

| **Species samples** | **Voucher/Catalog #** | **16S** | **ND4** | **RAG1** | **Locality** |
| --- | --- | --- | --- | --- | --- |
| *Rhampholeon spectrum* | MNHN 351I | AJ609599 | N/A | N/A | Cameroon, Mapanja, Mt. Cameroon |
| *Rhampholeon spectrum* | KU 348793 | OP716820 | OP734786 | OP734804 | Cameroon, Mt Cameroon |
| *Rhampholeon spectrum* | 179929 | N/A | AF443236 | N/A | Cameroon |
| *Rhampholeon spectrum* | KU 341891 | OP716836 | OP734770 | N/A | Cameroon, Korup Park |
| *Rhampholeon spectrum* | KU 341929 | OP716839 | OP734768 | OP734792 | Cameroon, Korup Park |
| *Rhampholeon spectrum* | KU 341930 | OP716841 | OP734765 | OP734793 | Cameroon, Korup Park |
| *Rhampholeon spectrum* | KU 341931 | OP716843 | OP734766 | N/A | Cameroon, Korup Park |
| *Rhampholeon spectrum* | KU 341932 | OP716838 | OP734769 | OP734794 | Cameroon, Korup Park |
| *Rhampholeon spectrum* | CAS 260179 | OP716842 | OP734767 | N/A | Cameroon, Korup Park |
| *Rhampholeon spectrum* | CAS 260180 | OP716844 | OP734771 | OP734791 | Cameroon, Korup Park |
| *Rhampholeon spectrum* | CAS 260131 | OP716837 | OP734763 | N/A | Cameroon, Korup Park |
| *Rhampholeon spectrum* | CAS 260132 | OP716840 | OP734764 | N/A | Cameroon, Korup Park |
| *Rhampholeon spectrum* | TNW101 | OP716826 | OP734784 | OP734806 | Cameroon, Mt. Kupe |
| *Rhampholeon spectrum* | TNW109 | OP716827 | OP734785 | OP734807 | Cameroon, Mt. Kupe |
| *Rhampholeon spectrum* | KU 348784 | OP716816 | OP734775 | OP734795 | Cameroon, Mt. Manengouba |
| *Rhampholeon spectrum* | KU 348785 | OP716817 | OP734776 | OP734796 | Cameroon, Mt. Manengouba |
| *Rhampholeon spectrum* | KU 348786 | OP716818 | OP734778 | OP734797 | Cameroon, Mt. Manengouba |
| *Rhampholeon spectrum* | KU 348787 | OP716819 | OP734777 | OP734798 | Cameroon, Mt. Manengouba |
| *Rhampholeon spectrum* | KU 348788 | OP716821 | OP734779 | OP734799 | Cameroon, Mt. Nlonako |
| *Rhampholeon spectrum* | KU 348789 | OP716822 | OP734783 | OP734800 | Cameroon, Mt. Nlonako |
| *Rhampholeon spectrum* | KU 348790 | OP716823 | OP734780 | OP734801 | Cameroon, Mt. Nlonako |
| *Rhampholeon spectrum* | KU 348791 | OP716824 | OP734781 | OP734802 | Cameroon, Mt. Nlonako |
| *Rhampholeon spectrum* | KU 348792 | OP716825 | OP734782 | OP734803 | Cameroon, Mt. Nlonako |
| *Rhampholeon spectrum* | CAS 207682 | OP716832 | OP734758 | OP734788 | Equatorial Guinea, Bioko Sur |
| *Rhampholeon spectrum* | CAS 207683 | OP716831 | OP734759 | OP734789 | Equatorial Guinea, Bioko Sur |
| *Rhampholeon spectrum* | CAS 207684 | OP716829 | OP734762 | N/A | Equatorial Guinea, Bioko Sur |
| *Rhampholeon spectrum* | CAS 207685 | OP716828 | OP734760 | OP734790 | Equatorial Guinea, Bioko Sur |
| *Rhampholeon spectrum* | CAS 207688 | OP716830 | OP734761 | AY524940 | Equatorial Guinea, Bioko Sur |
| *Rhampholeon spectrum* | MCZ 187706 | OP716834 | OP734774 | N/A | Gabon, Ogoue-Ivindo |
| *Rhampholeon spectrum* | MCZ 187707 | OP716833 | OP734772 | N/A | Gabon, Ogoue-Ivindo |
| *Rhampholeon spectrum* | MCZ 188637 | OP716845 | OP734773 | N/A | Gabon, Ogoue-Ivindo |
| *Rhampholeon spectrum* | PEM-R 16262 | AY524865 | N/A | AY524941 | Gabon, Mekambo |
| *Rhampholeon acuminatus* | CT 153 | HF570459 | N/A | N/A | Tanzania, Nguru Mtns |
| *Rhampholeon boulengeri* | UTEP 21715 | KM589410 | N/A | KM589416 | DRC, Itombwe Plateau |
| *Rhampholeon boulengeri* | UTEP 21716 | MG645841 | N/A | MG645931 | DRC, Itombwe Plateau |
| *Rhampholeon boulengeri* | CAS 201682 | AY524877 | N/A | N/A | Uganda, Ihihizo River |
| *Rhampholeon boulengeri* | CAS 201681 | AY524878 | N/A | AY524953 | Uganda, Ihihizo River |
| *Rhampholeon bruessoworum* | PEM-R 20374 | HG798975 | N/A | HG798999 | Mozambique, Mt. Inago |
| *Rhampholeon bruessoworum* | PEM-R 20375 | HG798976 | N/A | HG799000 | Mozambique, Mt. Inago |
| *Rhampholeon chapmanorum* | PEM-R 16245 | AY524881 | HF570596 | AY524956 | Malawi, Malawi Hill |
| *Rhampholeon chapmanorum* | PEM-R 17130 | EF114326 | N/A | N/A | Malawi, Malawi Hill |
| *Rhampholeon gorongosae* | PEM-R 16253 | AY524874 | N/A | AY524950 | Mozambique, Mt. Gorongosa |
| *Rhampholeon gorongosae* | PEM-R 16252 | AY524873 | N/A | AY524949 | Mozambique, Mt. Gorongosa |
| *Rhampholeon hattinghi* | PEM-R 19196 | KM589414 | N/A | N/A | DRC, Mt. Nzawa |
| *Rhampholeon hattinghi* | PEM-R 19197 | KM589415 | N/A | N/A | DRC, Mt. Nzawa |
| *Rhampholeon marshalli* | PEM-R 16243 | AY524870 | HQ130555 | AY524946 | Zimbabwe, Vumba Mtns |
| *Rhampholeon marshalli* | PEM-R 16244 | AY524871 | N/A | AY524947 | Zimbabwe, Vumba Mtns |
| *Rhampholeon maspictus* | PEM-R 17911 | HG798971 | N/A | HG798997 | Mozambique, Mt. Mabu |
| *Rhampholeon maspictus* | PEM-R 17912 | HG798972 | N/A | HG798998 | Mozambique, Mt. Mabu |
| *Rhampholeon moyeri* | MTSN 002TA | AY524875 | HF570598 | AY524951 | Tanzania, Udzungwa Mtns |
| *Rhampholeon moyeri* | MTSN 001TA | AY524876 | N/A | AY524952 | Tanzania, Udzungwa Mtns |
| *Rhampholeon nchisiensis* | PEM-R 16249 | AY524886 | HF570599 | AY524961 | Malawi, Nchisi Mtn |
| *Rhampholeon nchisiensis* | PEM-R 16242 | AY524883 | N/A | AY524958 | Zambia, Nyika Plateau |
| *Rhampholeon nebulauctor* | PEM-R 17281 | HG798974 | N/A | N/A | Mozambique, Mt. Chiperone |
| *Rhampholeon nebulauctor* | PEM-R 17280 | HG798973 | N/A | N/A | Mozambique, Mt. Chiperone |
| *Rhampholeon platyceps* | PEM-R 16251 | AY524879 | HQ130556 | AY524954 | Malawi, Mt. Mlanje |
| *Rhampholeon platyceps* | PEM-R 16250 | AY524880 | N/A | AY524955 | Malawi, Mt. Mlanje |
| *Rhampholeon spinosus* | CT118 | HF570460 | HF570600 | HF570779 | Tanzania, West Usambara Mtns |
| *Rhampholeon spinosus* | MHNG 2609.034 | AM055686 | N/A | N/A | Tanzania, West Usambara Mtns |
| *Rhampholeon temporalis* | PEMR16254 | AY524866 | HQ130553 | AY524942 | Tanzania, East Usambara Mtns |
| *Rhampholeon temporalis* | PEMR16255 | AY524867 | N/A | AY524943 | Tanzania, East Usambara Mtns |
| *Rhampholeon tilburyi* | PEMR17134 | EF114322 | N/A | EF114338 | Mozambique, Namuli Massif |
| *Rhampholeon tilburyi* | PEMR17135 | EF114323 | N/A | EF114339 | Mozambique, Namuli Massif |
| *Rhampholeon uluguruensis* | ZMB48421 | AY524896 | N/A | N/A | Tanzania, Uluguru Mtns |
| *Rhampholeon uluguruensis* | ZMB48431 | AY524897 | N/A | N/A | Tanzania, Uluguru Mtns |
| *Rhampholeon viridis* | PEMR16260 | AY524868 | N/A | AY524944 | Tanzania, North Pare Mtns |
| *Rhampholeon viridis* | PEMR16259 | AY524869 | N/A | AY524945 | Tanzania, South Pare Mtns |
| *Rhampholeon viridis* | CT204 | HF570461 | HF570602 | HF570780 | Tanzania, South Pare Mtns |
| *Archaius tigris* | ZSM 604 | HQ130515 | HQ130560 | HQ130624 | Seychelles |
| *Bradypodion gutturale* | KT047 | AY569793 | HF570525 | HF570726 | South Africa |
| *Bradypodion thamnobates* | PEM R5721 | AY289815 | HF570532 | HF570734 | South Africa |
| *Bradypodion ventrale* | KTH153 | AY756654 | HF570534 | HF570736 | South Africa |
| *Brookesia brygooi* | KUZ R61408 | HF570400 | FJ981799 | FJ984235 | Madagascar |
| *Brookesia lineata* | G33 | HF570404 | FJ981816 | FJ984252 | Madagascar |
| *Brookesia micra* | UADBA-FGZC 1831 | JN674050 | HF570538 | JN674062 | Madagascar |
| *Calumma boettgeri* | MVTIS 2002 | HF570416 | HM776661 | HF570739 | Madagascar |
| *Calumma hafahafa* | DRV 6282 | HF570438 | HF570561 | HF570762 | Madagascar |
| *Calumma linotum* | MVTIS 2001 | HF570424 | HF570546 | HF570748 | Madagascar |
| *Chamaeleo africanus* | MVZ 238898 | HF570439 | HF570562 | HF570763 |  |
| *Chamaeleo gracilis* | CT088 | FJ717748 | HF570568 | FJ746587 |  |
| *Chamaeleo namaquensis* | AMB5863 | HQ130516 | FJ981754 | FJ984185 | Namibia |
| *Furcifer labordi* | ZFMK 68616 | HF570450 | HF570575 | FJ984193 | Madagascar |
| *Furcifer pardalis* | ZSM 259 | HF570453 | HF570578 | HF570775 | Madagascar |
| *Furcifer verrucosus* | ZSM 40 | HF570458 | HF570581 | HF570778 | Madagascar |
| *Kinyongia carpenteri* | PEM R16572 | DQ923821 | HF570583 | DQ996660 |  |
| *Kinyongia tavetana* | PEM R5736 | DQ991233 | HF570590 | DQ996671 |  |
| *Kinyongia uthmoelleri* | PEM R165565 | HF570592 | DQ996674 | DQ923836 |  |
| *Nadzikambia baylissi* |  | HM582316 | N/A | HM582322 |  |
| *Nadzikambia mlanjense* | PEM R16294 | DQ923841 | HF570595 | DQ996679 |  |
| *Palleon nasus* | ZSM 728 | HQ130509 | FJ984202 | FJ981761 |  |
| *Rieppeleon brachyurus* | PEM-R 16263 | AY524898 | N/A | AY524968 | Tanzania, Near Tamota |
| *Rieppeleon brachyurus* | PEM-R 16264 | AY524899 | N/A | AY524969 | Tanzania, Near Tamota |
| *Rieppeleon brevicaudatus* | PEM-R 16256 | AY524887 | N/A | AY524962 | Tanzania, East Usambara Mtns |
| *Rieppeleon brevicaudatus* | PEM-R 16257 | AY524888 | N/A | AY524963 | Tanzania, East Usambara Mtns |
| *Rieppeleon kerstenii* | CAS 214834 | AY524891 | N/A | AY524966 | N/A |
| *Rieppeleon kerstenii* | CAS 169939 | AY524890 | N/A | AY524965 | Kenya, Kilifi |
| *Trioceros cristatus* | RMB 19416 | OP716845 | OP734787 | OP734805 | Cameroon, Korup Park |
| *Trioceros goetzei* | CT050 | FJ717768 | HF570610 | FJ746603 | Tanzania |
| *Trioceros johnstoni* | CT353 | FJ717776 | KJ908805 | FJ746610 |  |
| *Agama agama* | MCZ 184560 | JX668144 | JX857595 | N/A |  |
| *Physignathus cocincincus* |  | AB031990 | N/A | AY662582 | Thailand, Ko Chang |
| *Uromastyx acanthinura* |  | FJ639598 | N/A | AY662588 | Tunisia |

PEM = Port Elizabeth Museum, CAS = California Academy of Sciences, CT = field number of Colin Tilbury, MTSN = Museo Tridentino di Scienze Naturali, ZMB = Universität Humboldt, Zoologisches Museum, MHNG = Natural History Museum of Geneva, UTEP = University of Texas El Paso, MCZ = Museum of comparative zoology, KU = University of Kansas, MNHN = National Museum of Natural History, DRC = Democratic Republic of Congo, N/A = not applicable.
