# Supplementary material for: Diversification and historical demography of Rhampholeon spectrum in West-Central Africa
Source: PLoS One. 2022 Dec 16;17(12):e0277107. doi: 10.1371/journal.pone.0277107 (PMC9757597; doi:10.1371/journal.pone.0277107)
Supplement: S2 Table — (DOCX) [file pone.0277107.s007.docx]

**S2 Table.** Primers used for sequencing mitochondrial and nuclear genes.

| Primer Name | Sequence | Reference |
| --- | --- | --- |
| 16S(L2510) | 5'-CGCCTGTTTATCAAAAACAT-3' | Palumbi (1996) |
| 16S(H3080) | 5'-CCGGTCTGAACTCAGATCACGT-3' | Palumbi (1996) |
| ND4 | 5'-TGACTACCAAAAGCTCATGTAGAAGC-3' | Raxworthy et al. (2002) |
| tRNA^leu^ | 5'-CATTACTTTTACTTGGATTTGCACCA-3' | Raxworthy et al. (2002) |
| RAG1-ChamF1 | 5'-TTGGAAAACTACTTCCTGAAG-3' | Tolley et al. (2013) |
| RAG1-ChamF2 | 5'-GCCAACAGCAATAAAGGAGA-3' | Tolley et al. (2013) |
| RAG1(f335) | 5'-CCACTTGGAAAAATACTCCCTGA-3' | Townsend et al. (2009) |
| RAG1(r337) | 5'-GTCATCAACCAAATGTTGTATGCCTG-3' | Townsend et al. (2009) |
| RAG1-ChamR1 | 5'-CTATTGAGGATGTTCAGGAA-3' | Tolley et al. (2013) |
