# Supplementary material for: Diversification and historical demography of Rhampholeon spectrum in West-Central Africa
Source: PLoS One. 2022 Dec 16;17(12):e0277107. doi: 10.1371/journal.pone.0277107 (PMC9757597; doi:10.1371/journal.pone.0277107)
Supplement: S3 Table — Node letters correspond to those in Bayesian tree in supplemental S1 Fig. Posterior ages, in millions of years ago (Mya), are presented as median values and 95% confidence intervals. (DOCX) [file pone.0277107.s008.docx]

**S3 Table.** Divergence date priors for primary (fossil) and secondary calibrations. Node letters correspond to those in Bayesian tree in supplemental Figure S1. Posterior ages, in millions of years ago (Mya), are presented as median values and 95% confidence intervals.

| Node | Median in Myr (95% CI) | Source |
| --- | --- | --- |
| Primary Calibration |  |  |
| A | 101.7 (100.2-105.2) | Stem chameleon from Albian-Cenomanian  boundary, Cretaceous (Daza et al., 2016) |
| Secondary Calibration |  |  |
| B | 64.8 (63.2-66.4) | Node in Bayesian tree in Fig S1 (Tolley et al., 2013) |
| C | 53.9 (52.3-55.5) | Node in Bayesian tree in Fig S1 (Tolley et al., 2013) |
| D | 50.2 (48.6-51.8) | Node in Bayesian tree in Fig S1 (Tolley et al., 2013) |
| E | 48.3 (46.7-49.9) | Node in Bayesian tree in Fig S1 (Tolley et al., 2013) |
| F | 44.99 (43.1-46.9) | Node in Bayesian tree in Fig S1 (Hughes et al., 2018) |
| G | 36.1 (34.5-37.7) | Node in Bayesian tree in Fig S1 (Tolley et al., 2013) |
