# Supplementary material for: Diversification and historical demography of Rhampholeon spectrum in West-Central Africa
Source: PLoS One. 2022 Dec 16;17(12):e0277107. doi: 10.1371/journal.pone.0277107 (PMC9757597; doi:10.1371/journal.pone.0277107)
Supplement: S4 Table — (DOCX) [file pone.0277107.s009.docx]

**S4 Tables.** Votes out of 500 random forest classifiers for each of the 89 competing demographic models inferred in delimitR.

| **Models** | **1** | **2** | **3** | **4** | **5** | **6** | **7** | **8** | **9** | **10** |
| --- | --- | --- | --- | --- | --- | --- | --- | --- | --- | --- |
| **Votes received** | 0 | 0 | 3 | 0 | 1 | 3 | 0 | 0 | 3 | 6 |

| **Models** | **11** | **12** | **13** | **14** | **15** | **16** | **17** | **18** | **19** | **20** |
| --- | --- | --- | --- | --- | --- | --- | --- | --- | --- | --- |
| **Votes received** | 10 | 5 | 2 | 0 | 1 | 1 | 1 | 7 | 5 | 9 |

| **Models** | **21** | **22** | **23** | **24** | **25** | **26** | **27** | **28** | **29** | **30** |
| --- | --- | --- | --- | --- | --- | --- | --- | --- | --- | --- |
| **Votes received** | 15 | 0 | 0 | 0 | 3 | 1 | 5 | 1 | 3 | 8 |

| **Models** | **31** | **32** | **33** | **34** | **35** | **36** | **37** | **38** | **39** | **40** |
| --- | --- | --- | --- | --- | --- | --- | --- | --- | --- | --- |
| **Votes received** | 0 | 0 | 0 | 1 | 6 | 9 | 6 | 1 | 4 | 1 |

| **Models** | **41** | **42** | **43** | **44** | **45** | **46** | **47** | **48** | **49** | **50** |
| --- | --- | --- | --- | --- | --- | --- | --- | --- | --- | --- |
| **Votes received** | 1 | 0 | 2 | 5 | 14 | 10 | 13 | 2 | 1 | 1 |

| **Models** | **51** | **52** | **53** | **54** | **55** | **56** | **57** | **58** | **59** | **60** |
| --- | --- | --- | --- | --- | --- | --- | --- | --- | --- | --- |
| **Votes received** | 1 | 9 | 5 | 3 | 4 | 17 | 1 | 17 | 7 | 27 |

| **Models** | **61** | **62** | **63** | **64** | **65** | **66** | **67** | **68** | **69** | **70** |
| --- | --- | --- | --- | --- | --- | --- | --- | --- | --- | --- |
| **Votes received** | 4 | 1 | 12 | 1 | 11 | 12 | 3 | 11 | 3 | 11 |

| **Models** | **71** | **72** | **73** | **74** | **75** | **76** | **77** | **78** | **79** | **80** |
| --- | --- | --- | --- | --- | --- | --- | --- | --- | --- | --- |
| **Votes received** | 6 | 2 | 4 | 5 | 21 | 4 | 14 | 13 | 7 | 16 |

| **Models** | **81** | **82** | **83** | **84** | **85** | **86** | **87** | **88** | **89** |
| --- | --- | --- | --- | --- | --- | --- | --- | --- | --- |
| **Votes received** | 5 | 18 | 9 | 7 | 20 | 16 | 1 | 1 | 1 |
