# Supplementary material for: Diversification and historical demography of Rhampholeon spectrum in West-Central Africa
Source: PLoS One. 2022 Dec 16;17(12):e0277107. doi: 10.1371/journal.pone.0277107 (PMC9757597; doi:10.1371/journal.pone.0277107)
Supplement: S5 Table — (DOCX) [file pone.0277107.s010.docx]

**S5 Table.** Number of putatively unlinked SNPs used to produce the mSFS between populations for the six pairwise models used to test demographic scenario with ddRAD dataset2.

|  | Korup (n=10) | CCVL (n=12) | Gabon (n=4) |
| --- | --- | --- | --- |
| Bioko (n=8) | 2973 | 3878 | 1568 |
| Korup (n=10) | - | 4214 | 2103 |
| CCVL (n=12) | - | - | 2683 |
