# Supplementary material for: Diversification and historical demography of Rhampholeon spectrum in West-Central Africa
Source: PLoS One. 2022 Dec 16;17(12):e0277107. doi: 10.1371/journal.pone.0277107 (PMC9757597; doi:10.1371/journal.pone.0277107)
Supplement: S6 Table — Values along the diagonal indicate the number out of 10,000 simulated data sets that were correctly classified by the random forest classifiers. Model 1: no divergence, model 2: divergence without gene flow, model 3: divergence with secondary contact, and model 4: divergence with gene flow. (DOCX) [file pone.0277107.s011.docx]

**S6 Tables.** Confusion matrices from delimitR analyses. Values along the diagonal indicate the number out of 10,000 simulated data sets that were correctly classified by the random forest classifiers. Model 1: no divergence, model 2: divergence without gene flow, model 3: divergence with secondary contact, and model 4: divergence with gene flow.

| Confusion matrix : Bioko vs Korup | | | | |
| --- | --- | --- | --- | --- |
|  | Model 1 | Model 2 | Model 3 | Model 4 |
| Model 1 | 10000 | 0 | 0 | 0 |
| Model 2 | 0 | 9320 | 0 | 680 |
| Model 3 | 0 | 1 | 9999 | 0 |
| Model 4 | 0 | 2568 | 0 | 7432 |

| Confusion matrix : Bioko vs CCVL | | | | |
| --- | --- | --- | --- | --- |
|  | Model 1 | Model 2 | Model 3 | Model 4 |
| Model 1 | 9999 | 0 | 1 | 0 |
| Model 2 | 0 | 9324 | 0 | 676 |
| Model 3 | 3 | 1 | 9994 | 2 |
| Model 4 | 0 | 2547 | 0 | 7453 |

| Confusion matrix : Bioko vs Gabon | | | | |
| --- | --- | --- | --- | --- |
|  | Model 1 | Model 2 | Model 3 | Model 4 |
| Model 1 | 9998 | 0 | 2 | 0 |
| Model 2 | 0 | 8564 | 0 | 1436 |
| Model 3 | 10 | 3 | 9977 | 10 |
| Model 4 | 0 | 3413 | 0 | 6587 |

| Confusion matrix : Korup vs CCVL | | | | |
| --- | --- | --- | --- | --- |
|  | Model 1 | Model 2 | Model 3 | Model 4 |
| Model 1 | 9915 | 0 | 85 | 0 |
| Model 2 | 0 | 9360 | 0 | 640 |
| Model 3 | 479 | 0 | 9517 | 4 |
| Model 4 | 0 | 2719 | 0 | 7281 |

| Confusion matrix : Korup vs Gabon | | | | |
| --- | --- | --- | --- | --- |
|  | Model 1 | Model 2 | Model 3 | Model 4 |
| Model 1 | 10000 | 0 | 0 | 0 |
| Model 2 | 0 | 7813 | 0 | 2187 |
| Model 3 | 4 | 4 | 9987 | 5 |
| Model 4 | 0 | 3525 | 1 | 6474 |

| Confusion matrix : CCVL vs Gabon | | | | |
| --- | --- | --- | --- | --- |
|  | Model 1 | Model 2 | Model 3 | Model 4 |
| Model 1 | 10000 | 0 | 0 | 0 |
| Model 2 | 0 | 8739 | 0 | 1261 |
| Model 3 | 2 | 2 | 9992 | 4 |
| Model 4 | 0 | 3443 | 0 | 6557 |
