# Supplementary material for: Diversification and historical demography of Rhampholeon spectrum in West-Central Africa
Source: PLoS One. 2022 Dec 16;17(12):e0277107. doi: 10.1371/journal.pone.0277107 (PMC9757597; doi:10.1371/journal.pone.0277107)
Supplement: S7 Table — Model 1: no divergence, model 2: divergence without gene flow, model 3: divergence with secondary contact, and model 4: divergence with gene flow. (DOCX) [file pone.0277107.s012.docx]

**S7 Table.** Votes out of 500 random forest classifiers for each competing demographic model in delimitR. Model 1: no divergence, model 2: divergence without gene flow, model 3: divergence with secondary contact, and model 4: divergence with gene flow.

|  | **Model 1** | **Model 2** | **Model 3** | **Model 4** |
| --- | --- | --- | --- | --- |
| **Bioko vs Korup** | 0 | 0 | 500 | 0 |
| **Bioko vs CCVL** | 0 | 0 | 500 | 0 |
| **Bioko vs Gabon** | 132 | 15 | 243 | 110 |
| **Korup vs CCVL** | 1 | 16 | 412 | 71 |
| **Korup vs Gabon** | 169 | 10 | 301 | 20 |
| **CCVL vs Gabon** | 115 | 7 | 343 | 35 |
