# Supplementary material for: Diversification and historical demography of Rhampholeon spectrum in West-Central Africa
Source: PLoS One. 2022 Dec 16;17(12):e0277107. doi: 10.1371/journal.pone.0277107 (PMC9757597; doi:10.1371/journal.pone.0277107)
Supplement: S8 Table — (DOCX) [file pone.0277107.s013.docx]

**S8 Table.** DelimitR results for the pairwise interaction models.

|  | **Posterior probability** | **Error out of bag in %** | **Model selected** | **Number of vote out of 500** |
| --- | --- | --- | --- | --- |
| **Bioko-Korup** | 1 | 8.12 | 3 | 500 |
| **Bioko-CCVL** | 1 | 8.075 | 3 | 500 |
| **Bioko-Gabon** | 0.98 | 12.2 | 3 | 243 |
| **Korup-CCVL** | 1 | 9.82 | 3 | 412 |
| **Korup-Gabon** | 0.91 | 14.31 | 3 | 301 |
| **CCVL-Gabon** | 0.98 | 11.78 | 3 | 343 |
