# Supplementary material for: Diversification and historical demography of Rhampholeon spectrum in West-Central Africa
Source: PLoS One. 2022 Dec 16;17(12):e0277107. doi: 10.1371/journal.pone.0277107 (PMC9757597; doi:10.1371/journal.pone.0277107)
Supplement: S1 File — (DOCX) [file pone.0277107.s014.docx]

**Modified ddRADseq protocol:**

Genomic DNA was extracted from tissue samples preserved in 95% ethanol or RNAlater (Sigma-Aldrich) using the Maxwell RSC system (Promega). Genome-wide anonymous nuclear markers were sequenced for each individual following a ddRADseq protocol modified from Peterson et al. (2012). 300–500 ng of genomic DNA was double digested for each individual using the restriction enzymes *Sbf*I (restriction site 5’- CCTGCAGG-3’) and *Msp*I (restriction site 5’-CCGG-3’). The resulting products were bead-cleaned with AmpureXP beads (Agencourt) and individually barcoded using custom oligonucleotide adapters. A Pippin Prep (Sage Science, Beverly, MA) was used to size select pooled samples to a mean insert length of 541 base pairs (bp) (487–595 bp range) using internal standards. The resulting post-ligation products were amplified using a high-fidelity polymerase (Phusion, New England Biolabs) for eight cycles. An Agilent TapeStation was used to determine each pool’s final fragment size distribution and concentration. Library pools were combined in equimolar amounts for sequencing on one Illumina HiSeqX Lane (with a 10% *Phi*X spike-in and 150 bp paired-end reads).
